# Imidazole-Based Lithium Salt LiHDI as a Solid Electrolyte Interphase-Stabilising Additive for Lithium-Conducting Electrolytes

**DOI:** 10.3390/molecules29040804

**Published:** 2024-02-09

**Authors:** Marek Broszkiewicz, Bartosz Brzozowski, Tomasz Trzeciak, Aldona Zalewska, Jacek Ryl, Leszek Niedzicki

**Affiliations:** 1 Faculty of Chemistry, Warsaw University of Technology, Noakowskiego 3, 00-664 Warsaw, Poland; 2 Advanced Materials Center, Institute of Nanotechnology and Materials Engineering, Gdansk University of Technology, Narutowicza 11/12, 80-233 Gdansk, Poland

**Keywords:** lithium-ion, electrolyte, additive, SEI, conductivity, cycling

## Abstract

Lithium salt LiHDI (lithium 4,5-dicyano-2-(*n*-heptafluoropropyl)imidazolide) is proposed as a solid electrolyte interphase-stabilising additive for lithium-ion batteries, which can be added in a smaller amount than fluoroethylene carbonate (FEC) and vinylene carbonate (VC) additives. Electrolytes containing either lithium 4,5-dicyano-2-(trifluoromethyl)imidazolide (LiTDI) or battery-standard LiPF_6_ were tested with various amounts of LiHDI additive. Chemical stability in the presence of water and the thermal stability of LiHDI are on par with LiTDI. LiHDI additive does not negatively affect the properties of electrolytes. Conductivity measurements of solutions, galvanostatic cycling of graphite-LiFePO_4_ cells at room temperature, cells’ cycling at 60 °C, internal cell resistance monitoring during cycling, and XPS analysis of electrodes’ surfaces after cycling have been performed. LiHDI, unlike the FEC-VC mixture, does not negatively affect the properties of the electrolyte. Cycling showed improved capacity retention with LiHDI additive with both graphite and LiFePO_4_ as capacity-limiting electrodes over samples without additives. At elevated temperatures, samples with LiHDI exhibited better capacity retention during cycling than those with FEC-VC. Internal cell resistance can be correlated with capacity retention. XPS results show changes in the composition of SEI depending on the composition of the electrolyte and the duration of cycling.

## 1. Introduction

Lithium-ion cells have constantly been improving since their introduction in the early 1990s. Their dominance over the energy storage market has been undoubted for at least a decade now, especially in mobile devices and EV applications [1,2]. Even though materials for both electrodes are developed by countless teams all over the world, the field of electrolytes is not so thriving. One of the main obstacles to Li-ion cells’ further advance is their electrolyte, which is almost exclusively based on LiPF_6_ salt [3]. LiPF_6_ is known for high solubility in typical battery solvents, high conductivity [4], and reasonable compatibility with common electrodes [5,6]. However, its main flaws are poor thermal stability [7] and reactivity towards any amount of water, including trace moisture on the ppm level [8,9], or even towards carbonates’ decomposition products [10]. LiPF_6_ also has suboptimal or even no compatibility with new generations of electrodes, such as silicon-based anodes and cathodes or mixed-oxides cathodes [11,12]. A typical way to enhance the performance of the LiPF_6_ salt is to use additives that stabilise it, e.g., PF_5_ (first product of LiPF_6_ decomposition) stabilisers [13,14,15], water and HF (product of LiPF_6_ hydrolysis) scavengers [15,16], and SEI (solid electrolyte interphase) stabilisers [17,18,19,20]. Another approach is to use a completely new salt that would substitute LiPF_6_. However, for many years, despite multiple attempts by various groups, no salt has achieved a performance that would allow it to be used instead of LiPF_6_ and without any additives. Among the few that are close to that goal is LiTDI, i.e., lithium 4,5-dicyano-2-(trifluoromethyl)imidazolide (Formula S1) [21]. This salt is developed by our group, but it has also received growing interest from the battery community [22,23,24,25,26,27]. Among the main advantages of LiTDI over LiPF_6_ are good thermal stability, lack of HF generation [28], and perfect stability in water, even as a solution. It is also compatible with modern generations of electrodes (especially those not compatible with LiPF_6_) [12,29,30,31], all without sacrificing transport properties [32].

The most commonly used anodic material for lithium-ion batteries is graphite thanks to its low operating potential. The use of this anode requires the formation of a stable solid electrolyte interface (SEI). Various organic and inorganic compounds were reported as components of SEI layers, including LiF, Li_2_CO_3_, LiRCO_3_, R_2_CO_3_, Li_2_O, ROLi, and polymeric species like PEO [33,34,35]. The formation of a stable SEI requires the use of an additive as the interface formed with just electrolyte components does not provide sufficient stability [3]. Fluoroethylene carbonate and vinylene carbonate were established as good additives. Their beneficial influence is attributed to the formation of poly(VC) [36,37]. It has also been shown that the use of FEC leads to the formation of LiF [38], although it remains unclear if its presence is beneficial for graphitic anodes [39,40].

LiTDI was developed initially as one of three salts, with its two analogues, LiPDI (lithium 4,5-dicyano-2-pentafluoroethylimidazolide) and LiHDI (lithium 4,5-dicyano-2-(n-heptafluoropropyl)imidazolide) (Formula S2), containing two and three carbon perfluorinated sidechain, respectively. Our first work on this family of salts suggested unequal stability against lithium metal, with LiHDI being the most stable and longest lasting [41]. Even though its transport properties were not as good as LiTDI and LiPDI, the formation of the stable SEI layer, even at a very low concentration, predestines it to be a good material for the SEI-stabilising additive. The low effective concentration of an additive is obviously a requirement for economic reasons. However, it is also crucial not to change the transport properties of the original, unmodified electrolyte. Some commercially available additives do not meet this condition and are required to be added in quantities of as much as 10% or even more (FEC, for instance) [42,43,44,45,46]. For this reason, it is worth looking for alternative compounds.

Since the first article on the LiTDI family of salts (a member of which is LiHDI), it has been known that they are stable against moisture—in the air, for instance—and in the water environment, e.g., during synthesis. It also came up quickly that they can complex water molecules in a stable manner; that is, they not only keep water in the lithium cation solvation layer but actually form crystals with water in the crystalline structure. It is especially helpful during cycling by pulling and keeping water from doing harm to electrode components or from thickening the SEI layer. It was also shown by Xu et al. [25] that LiTDI might be a good electrolyte additive as it is able to keep the water from reacting with the LiPF_6_, stabilising the whole electrolyte and thus the whole cell. One may expect LiHDI to have similar traits as its structure is very similar to that of LiTDI in terms of charge distribution, active sites for cation coordination, etc. Structures of SEI created with LiTDI on both graphitic and silicon anodes were investigated. It was shown that the structure of such an SEI layer is significantly less crosslinked due to the suspension of the radical polymerisation of solvents [22]. The use of LiHDI as an SEI-stabilising additive is expected to improve crosslinking by creating higher-order radicals. Such radicals could not be stabilised in the same way as radicals created by defluorination of LiTDI, potentially permitting radical polymerisation of solvents to a greater extent and higher crosslink density.

Here, we present our latest results for LiHDI used as the SEI-stabilising additive for graphite anodes for Li-ion cells. The performance of LiHDI has been compared to the standard additives of FEC and VC. Most electrolytes were based on LiTDI, which provides superior stability to LiPF_6_. However, the LiPF_6_ electrolyte was also used as a reference for cycling. LiFePO_4_ has been chosen as a cathode for cycling. It is known for its stability and capacity retention, thus providing a system in which the cathode would not have a significant impact on the cells’ performance [47]. In such a system, the effect of components other than additives on cells’ performance should be minimised. The influence of additives on the cathode electrolyte interface was also investigated.

## 2. Results and Discussion

### 2.1. Conductivity

**Figure 1:**
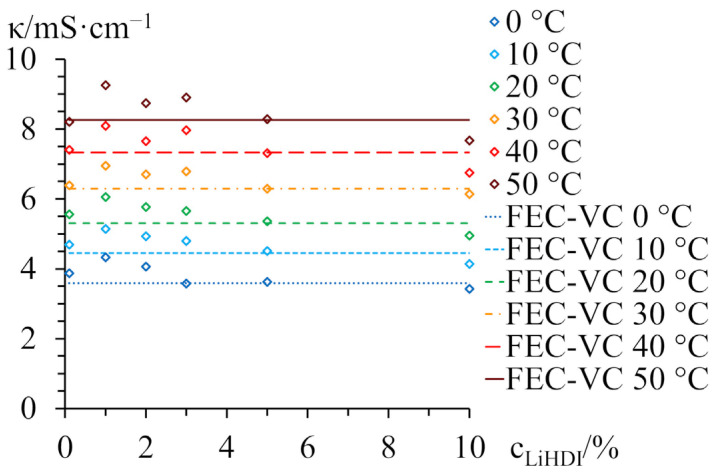
Conductivity of 0.63 mol·kg^−1^ LiTDI EC:2DMC electrolytes with various additives. The conductivity values of a reference electrolyte with 10% FEC and 2% VC are given as vertical lines.

Figure 1 exhibits the conductivity of a pristine 0.63 mol·kg^−1^ LiTDI electrolyte, as well as 0.63 mol·kg^−1^ LiTDI-based electrolytes containing 1%, 2%, 3%, 5%, and 10% LiHDI. Conductivities of 0.63 mol·kg^−1^ LiTDI with 10% FEC and 2% VC are also shown (as a horizontal line) as a reference. It is visible that the addition of LiHDI does not negatively affect ionic transport properties. Pristine 0.63 mol·kg^−1^ LiTDI has an ionic conductivity value of 5.6 mS·cm^−1^ at 20 °C. The 1% LiHDI addition slightly improves it (to 6.1 mS·cm^−1^); electrolytes with 2–5% LiHDI content show conductivity value on the same level (within 5.4–5.8 mS·cm^−1^); and 10% LiHDI addition has a slightly lower conductivity value (5.0 mS·cm^−1^), although all conductivity values are within a 5% spread. In terms of conductivity, the 1–3% LiHDI content range seems to be the most advantageous. Further increasing the additive content does not seem to be helpful, although it is not harmful either. The overall conductivity of these electrolytes is higher than the conductivity of the electrolyte containing FEC and VC, with the exception of the electrolyte with 10% LiHDI content.

The additional calculations of ionic conductivity process activation energy for electrolytes at each LiHDI concentration are in concert with the most advantageous conductivities. The pristine electrolyte and the 1–2% LiHDI content electrolytes possess the activation energy values at the same level (~11.5 kJ·mol^−1^). Electrolytes with 3% and higher LiHDI content have visibly higher activation energy (in the 12.6–13.1 kJ·mol^−1^ range). This might suggest a slightly different conductivity mechanism or different ion environment. It might further be explained by the concentration of available lithium cations in the system, as was explained before [32]. In this case, 0.63 mol·kg^−1^ LiTDI concentration is close to the 3:1 ratio between Li^+^ cations and solvating EC molecules. For this salt concentration range, the fraction of EC molecules involved in the lithium cation solvation layer formation is close to 50% of the total EC molecules in the solution. As the weight ratio between EC and DMC is 1:2, this means that there is an exactly 6:1 ratio between EC molecules (Mw = 88 g·mol^−1^) and lithium salt at the 0.63 mol·kg^−1^ concentration. The addition of LiHDI salt contributes to the higher lithium cation concentration in the solution. In the case of LiHDI, 1% content is slightly over 0.04 mol·kg^−1^, so 2% is equal to 0.08 mol·kg^−1^, 3% is roughly 0.12 mol·kg^−1^, 5% is around 0.21 mol·kg^−1^, and 10% is 0.41 mol·kg^−1^. This means that as close as 3% LiHDI content, the ratio between lithium cations and total EC molecules in the solution drops below 5:1 (4.85:1 to be exact), and it ends at 3.63:1 for 10% LiHDI content. One may speculate that the sole change in this ratio may be responsible for a change in the lithium cation solvent layer composition and a change in the overall lithium cation transport. One should also remember that LiHDI is likely to exhibit different associations than LiTDI, which results in a shift of optimal salt concentration. As LiHDI has a small or close to negligible effect on conductivity, it might be investigated for its impact on SEI formation and SEI stability during cycling. For the subsequent experiments, 2% and 3% of LiHDI additive are used, as higher content is not justified considering electrolytes’ transport properties. The lower content of the additive is also desirable from an economic point of view. However, 1% of LiHDI additive may not passivate the lithium metal sufficiently, as has been observed in the previous, preliminary findings (Figure S45).

### 2.2. Cyclic Voltammetry

**Figure 2:**
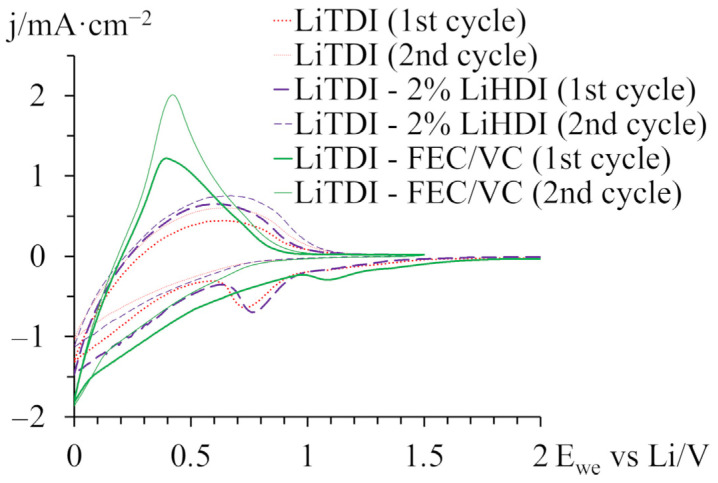
Voltammograms of (−) Li|electrolyte|graphite (+) system with three electrolytes presenting the first and the second cycles.

Figure 2 shows voltammograms of samples containing electrolytes without additives, with 2% of the LiHDI and with both 10% FEC and 2% VC. Plots show the first cycles of measurements. On these plots, we can see the current used for the formation of SEI layers. A signal is observed ca. 1–1.2 V vs. Li 0.63 mol·kg^−1^ LiTDI with FEC/VC additives, 0.7–0.9 V vs. Li for 0.63 mol·kg^−1^ with 2% LiHDI content, and 0.6–0.8 V vs. Li for 0.63 mol·kg^−1^ LiTDI without additives. Below this potential, we can see a current related to the intercalation of lithium cations into graphite. The signal related to the formation of the SEI layer is not visible in the subsequent cycles. It is important that reactions involving LiHDI occur at higher potentials than those with LiTDI; it means that LiHDI can potentially be used in LiTDI-based electrolytes as an SEI-stabilising additive.

### 2.3. Galvanostatic Cycling at 25 °C

**Figure 3:**
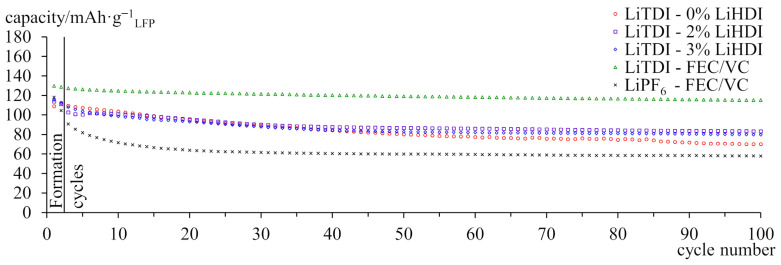
Capacity of graphite-LiFePO_4_ cells cycled at 25 °C with C/5 current. A cathode is the capacity-limiting electrode. The specific capacity was calculated per gram of the cathode’s active material.

Figure 3 shows the discharge capacity of full cells with equal-sized electrodes over 100 cycles. A sample containing LiPF_6_ was used as a reference. Samples with FEC/VC exhibit higher initial capacity than samples with LiHDI or without additives. The capacity of the sample containing LiPF_6_ drops to ca. 50% within the first 20 cycles and remains stable for the rest of the experiment. The sample containing LiTDI with FEC/VC exhibits the lowest decrease in capacity. Samples containing the LiHDI additive show improvement in capacity retention over pure LiTDI electrolyte, and the sample with 2% of the additive yields better results than the sample with 3% additive content. LiHDI did not provide as high a capacity as FEC/VC with LiTDI. However, the sample with FEC/VC is constantly losing capacity even after 100 cycles. It results in 87.7% capacity retention for 0.63 mol·kg^−1^ LiTDI with FEC/VC and 72.2% retention for 0.63 mol·kg^−1^ LiTDI with 2% LiHDI additive.

**Figure 4:**
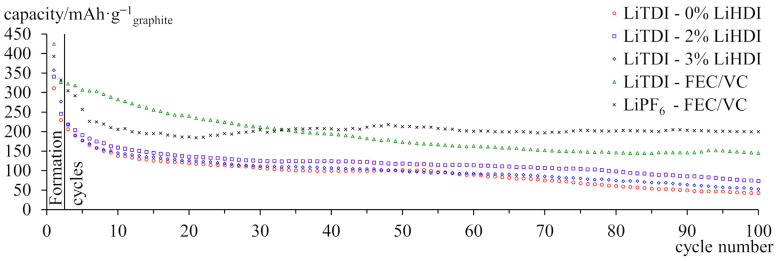
Capacity of graphite-LiFePO_4_ cells cycled at 25 °C with C/5 current. An anode is the capacity-limiting electrode. The specific capacity was calculated per gram of anode’s active material.

Figure 4 shows the discharge capacity of full cells with graphite as the capacity-limiting electrode. All of the samples exhibit significant capacity loss. Samples containing LiHDI performed better than the sample without additive, while the sample with 2% LiHDI retained more capacity than the sample with 3% of the additive. Samples containing FEC and VC have lost less capacity than other cells. Although capacity significantly fades in the first few cycles, capacity loss slowed down and was even partially reversed for the sample containing LiPF_6_. For the subsequent, more in-depth experiments, we have selected only electrolytes based on LiTDI for easier comparison between the samples. The experiment involves those containing commercial additives, 2% of in-house LiHDI, and the electrolyte without additives as a reference.

**Figure 5:**
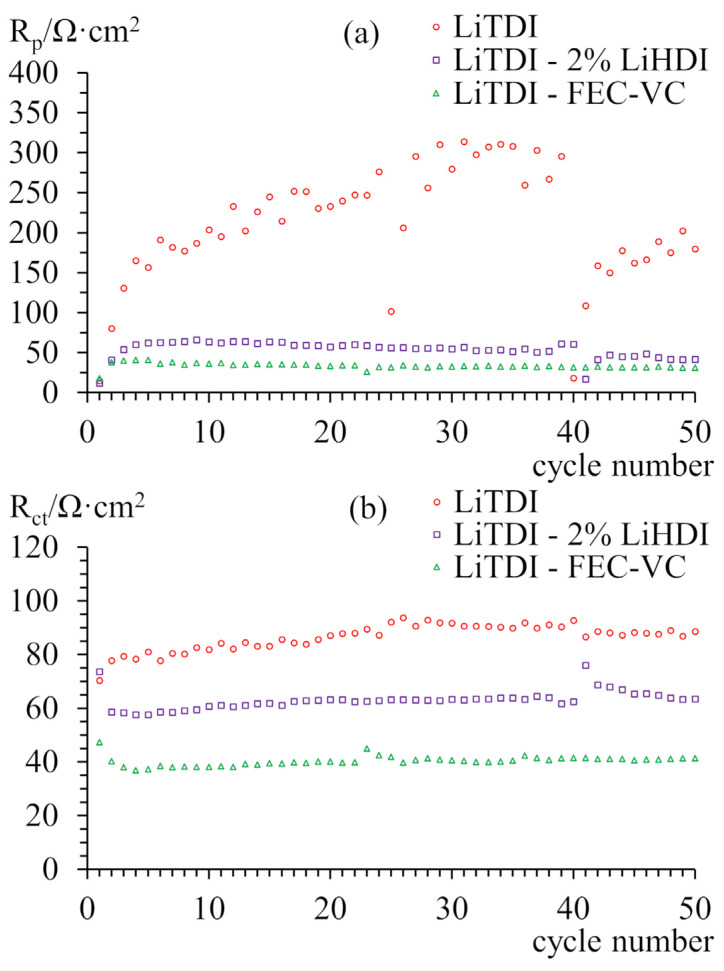
Resistances of SEI layers (**a**) and charge transfer (**b**) measured for graphite-LiFePO_4_ cells with a cathode as the capacity-limiting electrode. Resistances were measured in the charged state.

Figure 5a presents a comparison of passivation layer resistances obtained from impedance spectra over the first 50 cycles normalised with respect to the surface of the electrodes. Samples containing additives exhibit similarly low resistance of ca. 40–50 Ω, even slightly decreasing, throughout cycling. A significant increase in this resistance can be observed only in the first cycles for both samples. The sample that does not contain additives behaves differently. Although it has a similar initial resistance of the passivation layer as the other samples, it rapidly grows to over 200 Ω and exhibits great variation in subsequent cycles. Resistances of charge transfer are presented in Figure 5b. These resistances do not change significantly during cycling and are akin to each other for every sample.

**Figure 6:**
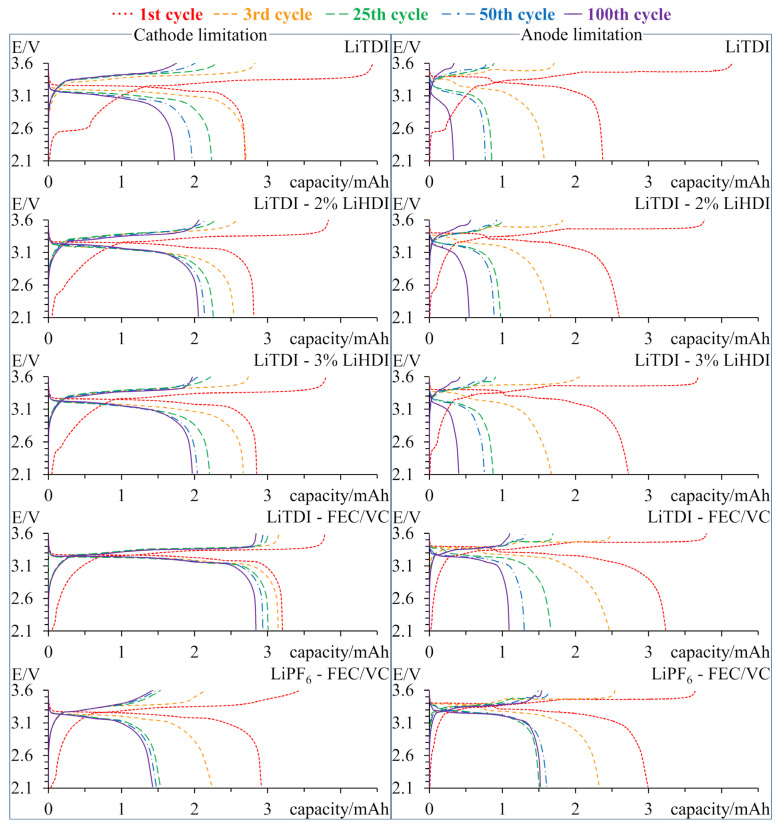
Charge–discharge curves of 1st, 3rd, 25th, 50th, and 100th cycles for samples cycled with various electrolytes. On the left, there are samples with a cathode as the capacity-limiting electrode; while on the right, there are samples with an anode as a capacity-limiting electrode.

Figure 6 shows the comparison of charge–discharge curves of the 1st, 3rd, 25th, 50th, and 100th cycles. The left-side plots show curves for cells with the cathode as the capacity-limiting electrode, while the right side is for cells with graphite as the capacity-limiting electrode. In the first cycle, we can see the decomposition of the electrolyte related to the formation of SEI. Decomposition is more significant for samples without additives. In the subsequent cycles, we can see a decrease in capacity resulting from faster cycling accompanied by a slight increase in polarisation. Samples with graphite limitation exhibit lower capacity than samples with cathode limitation, which, considering that the anode has a much higher specific capacity, is a clear indication that desired electrodes are indeed limiting capacity. Only samples with LiPF_6_ have similar capacities. Graphite-limited samples show a much higher capacity loss. Samples with LiPF_6_ are again an exception. On the curves of those samples, we can see plateaux corresponding with different stages of graphite intercalation. On the curves of cathode-limited samples, we cannot see all of the plateaux due to the fact that not all of the anodes’ capacity is being used. We can observe significant differences in the evolution of charge curves for anode-limited samples. The behaviour of samples with LiHDI is similar to that of the sample without additives. Samples with FEC/VC behave differently. Unlike samples with LiHDI, the capacity loss does not evenly affect all stages of intercalation. Capacity is lost mostly from the highest potential stage. For the sample containing LiPF_6_, a decrease in capacity in the 3.4–3.5 V region is coupled with an increase in capacity in the 3.3–3.4 V region.

There are many reasons for the capacity loss of a lithium-ion cell that can be attributed to the degradation of the anode or the cathode or decomposition of the electrolyte. Decomposition of the electrolyte can result in an increase in the internal cell’s resistance. An increase in charge transfer resistance or passivation layer resistance on any of the electrodes due to changes in the SEI layer can also influence polarisation and cause capacity loss. Degradation of the active material of electrodes is the main capacity-limiting effect on the anodes’ side [48]. Based on Figure 5b, we can state that the resistance of the charge transfer does not change significantly during cycling and between samples, regardless of capacity performance. Only passivation layer resistance changes significantly during cycling for the sample without additives. Also, looking at the charge–discharge curves allows us to rule out electrolyte decomposition as a culprit of capacity loss. We cannot see a significant increase in polarisation, even for samples without additives. However, the effect of increased internal resistance might have a more significant impact during faster cycling.

Looking at the first cycles presented in Figure 6, we can see that the addition of LiHDI significantly reduced the charge used for irreversible reactions. The charge used for these reactions is generally smaller for cells with smaller anodes as it is dependent on the anode’s surface. It proves that LiHDI forms a passivation layer on the graphite surface, preventing further electrolyte decomposition.

Based on the impedance data, we can say that LiHDI possesses similar SEI layer resistance stabilisation properties as FEC and VC. The main difference between these additives can be seen in the charge–discharge curves. The main reason for capacity loss related to the cathode material is the loss of lithium on side reactions, which include the consumption of lithium cations for SEI formation and may also include the deposition of metallic lithium on the anode. Another process is the dissolution of phosphates in the electrolyte [49]. Judging by the lack of changes in the number of plateaux observed for the samples with cathode limitation, we can say that the cathode-related process is responsible for the capacity loss. It, in turn, gives us information about the amount of lithium lost for the SEI formation. In this case, we can see that additives prevent the continued growth of the SEI layer, as evident in the increased capacity retention for samples with additives. We can also notice a slight decrease in polarisation for samples with LiHDI in contrast to a slight increase in polarisation for the sample without additives. It is in good correlation with impedance results. We can see that SEI created with FEC-VC uses less lithium over 100 cycles than the one created with LiHDI. What is interesting is that the sample containing LiPF_6_ as the main salt creates SEI that uses much more lithium. This is probably related to the decomposition of PF_6_^−^, resulting in increased content of LiF in the SEI layer [50]. It happens regardless of the presence of additives. For samples with graphite as a capacity-limiting electrode, we can see that FEC and VC additives are better at preventing anode degradation. It might be due to the higher quantity of additives allowing for a continuation of SEI reconstruction after its inevitable degradation. The second explanation might be the different composition of the SEI layer, which provides better protection from degradation. It is also interesting that capacity loss occurs mostly during the first stage of intercalation. Such behaviour can be seen for samples containing FEC and VC, regardless of the primary salt used in the electrolyte. It suggests progressive blocking of the first stage of graphite intercalation by the SEI layer. It is detrimental to the cell performance as it guarantees at least 50% capacity loss of the anode’s active material. This phenomenon is not observed for other samples, which presents the possible advantage of different additives over FEC and VC.

### 2.4. XPS Studies

Figure 7 shows XPS depth profiles of electrodes after formation cycles and 50 cycles with three electrolytes: LiTDI without additives, LiTDI with 2% LiHDI, and LiTDI with FEC/VC. Profiles of anodes are presented on the left side, while profiles of cathodes are shown on the right side. Plots show the relative content of lithium, carbon, nitrogen, oxygen, fluorine, phosphorus, and iron atoms at the given depth (measured in etching time). Table S1, presenting an assignment of the XPS signals, is provided in the Supplementary Materials. The depth profiles showing the relative content of the aforementioned elements with the distinction of their chemical environments are also presented in the Supplementary Materials in Figures S4–S44.

Analysis of anode profiles for the electrolyte without additives shows that after formation cycles, carbon content begins at less than 20% and starts to increase after 200 s of etching to constitute over 90% of the sample after 9000 s. The content of fluorine constitutes less than 10% of the sample and has a maximum after 100 s of etching. The content of lithium initially increases slightly from the initial 46% and subsequently decreases, ending with almost 0%. Fully lithiated graphite corresponds to the LiC_6_ structure. It corresponds to ca. 14% lithium atomic content. Due to the fact that cells were cycled with a cathode as a capacity-limiting electrode, the lithiation level at the end of the cycle was ca. 40%, which corresponds to ca. 5% atomic content [51].

The observed content deep into the sample is in a similar range, while its share at the external SEI part is much higher and results from electrolyte decomposition. The content of oxygen follows a similar pattern as lithium, but its content is much lower. The content of nitrogen is negligible. The cycled sample shows similar patterns; however, values are significantly shifted. Carbon content is about three times higher at the surface and increases significantly after only 20 s of etching. Contents of oxygen and lithium are much lower. The maximal content of lithium is observed after 10 s as opposed to 100 s for the first sample. After 50 cycles, we can also see more nitrogen in the sample, especially close to the surface.

The sample cycled with LiHDI additive shows a similar profile after formation cycles to that of the sample without additives; however, the carbon content is slightly lower, while the fluorine content is higher. It is also characterised by a decrease in carbon content with a depth of SEI. The minimum of carbon content corresponds well with the maximum of lithium content. After 50 cycles, the profile shows similar features. Akin to samples without additives, features are shifted closer to the surface. A significant increase in the content of oxygen is also noteworthy. Both samples with LiHDI contain a very small amount of nitrogen.

Samples containing FEC and VC behave slightly differently. The content of lithium shows a clear maximum for both samples. Similarly, carbon exhibits a minimum of its content. The content of oxygen is relatively low for the sample after formation cycles and increases after 50 cycles. On the other hand, fluorine content is quite high after formation cycles and significantly decreases after 50 cycles. The nitrogen content is very low in both samples.

All samples of cathodes show much less variation than anodes. The main differences are limited to the contents of carbon and fluorine. Starting from elements that do not vary significantly between samples, contents of phosphorus and iron start at almost 0% and slowly increase to ca. 20% each (which corresponds to their approximate content in LiFePO_4_). Oxygen contents follow a similar pattern, although they are always higher than the phosphorus and iron contents. This makes sense considering the fact that these elements constitute the cathode’s active material. However, the contents of oxygen do not represent the stoichiometry of FePO_4_, never exceeding 32%. There is one more noteworthy aspect of oxygen content: for the sample without additives after 50 cycles, it is much higher close to the surface, exceeding 10% as opposed to ca. 5% for other samples. It is related to the signal at 531.1 eV, which can be attributed to oxygen in carbonates. This signal is in close proximity to another signal at 533.2 eV, related to PO_4_, which can lead to a false increase in the weaker signal, especially deeper in the sample. Nitrogen contents are very small, reaching 3–6% close to the surface and quickly decreasing with depth. They are slightly higher for samples without additives and for the sample with 2% of the LiHDI after 50 cycles.

Profiles of cathodes show the highest content of carbon, which contributes over 60% of the sample. It also decreases with the depth for every sample, although the depth at which it starts to decrease varies between 100 and 600 s of etching. Such a high content of carbon is justified by the carbon coating of LiFePO_4_ grains. As all samples were treated in the same manner; differences coming from potential adventitious carbon should be negligible, especially considering the relatively low carbon content close to the surface for the samples of anodes. Also, the lack of electrolyte and charge transfer resistances change during cycling makes variations resulting from different electrolyte penetrations of the cathode highly unlikely. Thus, variation in carbon content is probably the result of content changes of other elements, mainly fluorine.

Fluorine constitutes ca. 15–20% of a sample and slowly decreases with depth to ca. 5–7%. The amount of fluorine increases for every electrolyte during cycling. The highest content of fluorine is observed for the sample without additives. Samples with 2% of the LiHDI exhibit a similar profile with a slightly lower amount of fluorine, while samples with FEC and VC show a higher content of fluorine on the surface coupled with a faster decrease with depth.

When we take a look at the evolution of SEI layer composition on graphite anodes, we can see a drastic change in behaviour between samples with and without additives. Unsurprisingly, additives stabilise the SEI layer, resulting in a much lower variation of composition. An interesting point of information about the XPS spectrum of the sample without additives after 50 cycles is that the very high carbon content shows closer to the surface, suggesting the exposition of the active material. Although we can also see an increase in the amounts of fluorine and nitrogen related to the decomposition of salt, it is nowhere near sufficient to explain the contents of carbon. It probably means that SEI in the sample without additives is cracked, which would correlate well with the variation of resistance observed in the impedance studies. For the samples without additives and containing LiHDI, a significant part of carbon content close to the surface after formation can be attributed to the signal at 289.6 eV, which might correspond to CO_3_ moiety [52]. Those samples measured after 50 cycles do not exhibit the presence of carbonates. On the other hand, samples containing FEC and VC showed an increase in the quantity of carbonates in SEI during cycling.

For samples with LiHDI, we can see a decrease in the content of oxygen, while for FEC/VC, the content of oxygen increases. The reverse tendency can be seen for fluorine. At the same time, we can see an increase in the content of lithium for samples with FEC/VC and a decrease for samples with LiHDI. It is interesting that for samples without additives and for samples with LiHDI, changes to SEI composition are observed after a shorter etching time after 50 cycles in comparison to samples after formation. For samples containing FEC and VC, the reverse is true. The thickness of the SEI layer can be estimated by the etching time at which carbon content drastically increases. It seems like the depth of the SEI is increasing during cycling only for samples with FEC and VC, while it is decreasing for the others. This is surprising, considering that FEC/VC produces the SEI layer of the most stable resistance. It might be that the resistance of SEI created with FEC and VC is much lower and does not change significantly with its thickness. It would also suggest that the resistance of SEI created with LiTDI is much higher despite it being very thin. It might also be the case that SEI created with LiTDI is forming, cracking, falling off, and then forming again. However, SEI formed with LiHDI also seems to be thin, while its resistance remains low and does not vary during cycling, which would favour the first explanation of LiTDI behaviour. This is also supported by the fact that there are no significant electrolyte resistance changes that would occur if continued LiTDI consumption on SEI formation took place. Based on this, we can speculate that the high content of oxygen for FEC- and VC-created SEI comes from the decomposition of additives and is responsible for better prevention of graphite decomposition. Decreasing contents of fluorine suggest that products of FEC decomposition, which are the source of fluorine, are soluble in the electrolyte. Otherwise, the amount of fluorine would increase. Proposed decomposition paths of FEC indicate HF and VC or fluorinated polycarbonates as products. In the case of HF generation, we should observe significant corrosion coupled with LiF deposition in the SEI layer [39]. The solubility of LiF in DMC is very small; however, in EC, much higher solubility was reported [40]. The high solubility of LiF in comparison with other components of SEI can explain the observed behaviour. The solubility of LiF in the electrolyte is sufficient to dissolve the amount of LiF of the same order of magnitude as the amount that could be created, at most, with 10% of the FEC additive [53]. Considering samples with LiHDI, we can see a significant increase in fluorine content, which can only come from salt or additive decomposition. It would mean that salts are continuously decomposing during cycling. It is worth noticing, however, that for all samples, the vast majority of fluorine is related to the same signal at 685.1 eV, which can be attributed to LiF [52]. In conjunction with the fact that the content of other fluorine-containing species does not significantly vary and remains at the level, which can be explained by the presence of PVdF binder, it is convincing that HF and VC are the main decomposition products of FEC. When we take this into consideration, we come to the conclusion that either salt and additive decompose to a greater extent than for the FEC/VC samples or dissolution of LiF is significantly hindered. Another interesting observation is that an increased amount of LiHDI additive, which would result in higher fluorine content, does not have a beneficial impact on the performance of graphite-LiFePO_4_ cells. It would seem oxygen, not fluorine, plays a crucial role in stabilising SEI on the graphite anode, resulting in better performance of FEC/VC samples with graphite as the capacity-limiting electrode. The role of LiF in SEI properties is unclear. Some researchers point out the fast surface diffusion of lithium cations and distortion in poorly conducting polymeric SEI as proof of its beneficial impact, while others attribute poor properties and capacity degradation to its low bulk conductivity and insufficient mechanical resistance [39,40]. Our results suggest that the lower content of LiF improves the performance of graphite anodes. An increase in the amount of lithium bound in SEI seems to have a positive effect on the cell’s performance. On the other hand, we can notice that lithium loss on SEI formation would negatively affect cells by its entrapment and thus the decrease in capacity in cells with the cathode as a capacity-limiting electrode.

Looking at the profiles of cathodes for samples, we can see a slight increase in the content of fluorine during cycling. Unfortunately, based on those results, we cannot state whether it is beneficial or not, as regardless of which electrode limits the capacity, the capacity loss of the cathode is related to the process taking place at the anode (loss of lithium for side reactions). It is also worth noticing that unlike anodes, where the majority of fluorine is in the form of LiF, for cathodes, the majority of fluorine is in the form of PVdF, as suggested by the presence of two signals at 686.3 eV and 688.2 eV [54,55]. The pair of signals is not observed for the anodes, probably due to the lower content of PVdF in graphite electrodes and thus the lower relative intensity of those signals.

### 2.5. Galvanostatic Cycling at 60 °C

**Figure 8:**
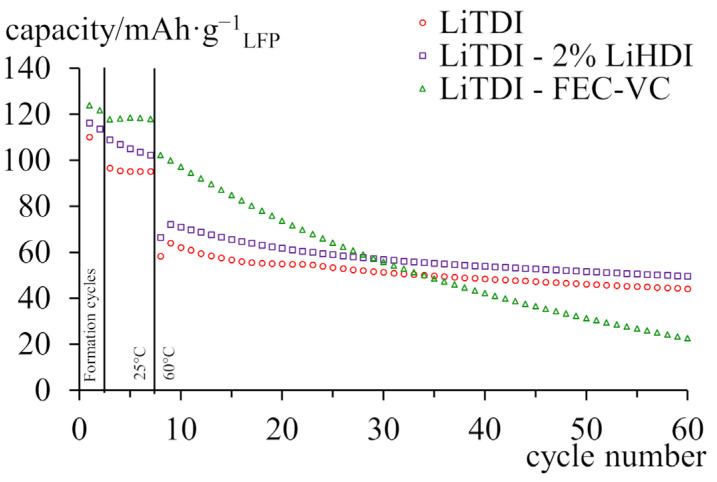
Capacity of graphite-LiFePO_4_ cells cycled at 60 °C with C/5 current. A cathode is the capacity-limiting electrode. The specific capacity was calculated per gram of the cathode’s active material.

Finally, to present the practical performance of additives in compositions, cycling at an elevated temperature has been carried out. Figure 8 shows the results of galvanostatic cycling at 60 °C. All samples exhibit similar capacities when cycled at 25 °C, both showing a slow decline. After samples were heated to 60 °C, the capacity of the sample containing FEC/VC decreased slightly, which was followed by a faster decline. However, the capacity of the sample with LiHDI immediately decreased to a similar value, at which point it stabilised during cycling at 25 °C. Sample containing LiHDI exhibits great stability during cycling at an elevated temperature. After 30 cycles, the capacity of the sample with FEC and VC drops below the capacity of the sample with LiHDI. The sample containing LiHDI shows a similar improvement in capacity over the sample without additives both in the room and at elevated temperatures.

**Figure 9:**
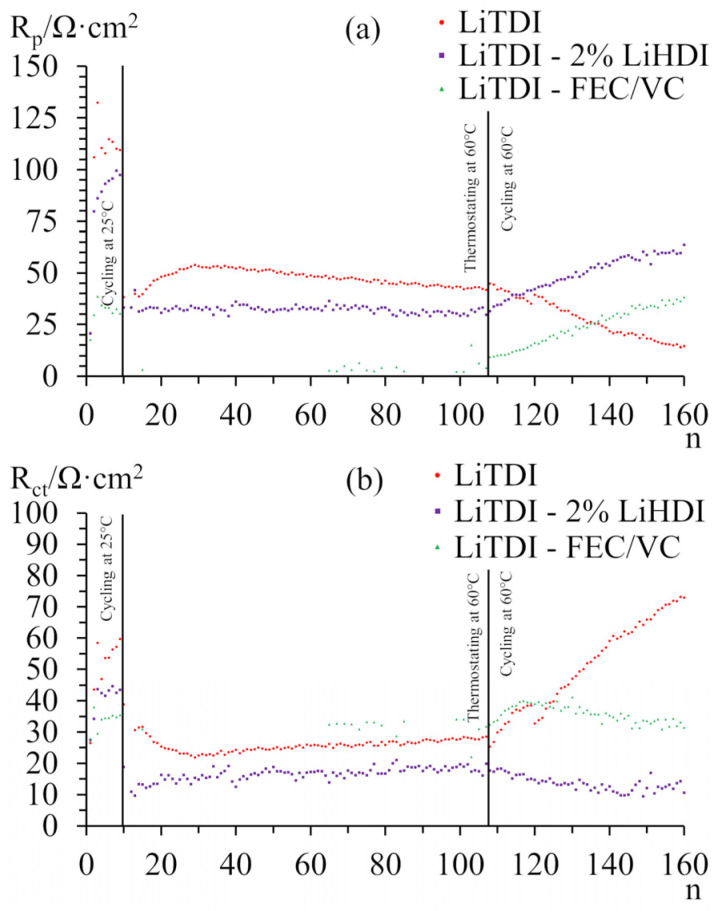
Resistances of SEI layers (**a**) and charge transfer (**b**) measured for graphite-LiFePO_4_ cells with a cathode as the capacity-limiting electrode. Resistances were measured in the charged state during cycling at 25 °C, thermostating at 60 °C, and cycling at 60 °C.

Figure 9 presents the results of the impedance analysis of the samples cycled at the elevated temperature. Figure 9a shows the evolution of passive layer resistance during cycling at 25 °C, thermostating at 60 °C, and cycling at 60 °C. Figure 9b presents the evolution of resistances of charge transfer. The initial behaviour of samples is similar to the samples cycled at 25 °C, both in terms of passivation resistance and charge transfer resistance. During thermostating, both resistances immediately decrease for all samples. Only the sample without additives shows notable changes in resistances. During cycling at the elevated temperature, the sample without additives shows a reduction of passivation layer resistance, while samples with additives show an increase in this resistance at the same and constant rate. The charge transfer resistance, however, increases significantly for the sample without additive while decreasing slightly for the other two. It should be noticed that the total internal resistance of samples increases at the same rate for all the samples. The sample with LiHDI shows a similar change in the resistances as the sample with FEC-VC and almost identical total resistance. It is a more favourable result for LiHDI in comparison to the resistances at 25 °C. However, it is not sufficient to explain the far worse performance of the sample with FEC-VC. The immediate decrease in the capacity for all the samples after an increase in the temperature suggests that the evolution of the SEI layer took place during heating. Such a change, however, is not reflected in changes in resistance. The lack of such correlation suggests that in the given conditions, the observed variation in resistance of the cell does not impact its capacity. The capacity evolution of the sample containing FEC and VC suggests that increased temperature does not, in itself, cause degradation of SEI; however, cycling caused much faster deterioration. It is also possible that FEC undergoes faster decomposition at higher temperatures, producing LiF, which, as mentioned earlier, may negatively impact cell performance. Defluorination of FEC was proposed either in the presence of Lewis acids [56] or electrochemically [38]. Without LiPF_6_ in the electrolyte, the first option is unlikely. However, at a higher temperature, the dissolution of SEI might be expedited, causing faster FEC decomposition. Such a process was proposed in the literature [57]. The loss of lithium for the formation of LiF can explain the observed loss of capacity, considering the content of FEC in the cell. Based on this result, we can say that LiHDI has superior performance at elevated temperatures.

### 2.6. Thermal Analysis

The thermal stability of electrodes, electrolytes, and electrolytes in contact with electrodes was investigated by DSC. Results are shown in Figure S2. None of those systems showed any sign of the thermal effect of the reaction despite the fact that some evolution of the system clearly occurred, at least for the electrolytes containing LiHDI. No signal was seen, even during the measurement of isothermal DSC at 60 °C.

The comparison pictures of electrolytes with LiHDI and FEC-VC additives before and after storage at 60 °C were also presented (Scheme S1). The slight change of colour of the FEC-VC-containing sample confirms the instability of those additives at higher temperatures, even in the absence of LiPF_6_. It does not, however, explain the behaviour of cells with LiHDI additive.

## 3. Materials and Methods

LiTDI and LiHDI were synthesised using a method that was described previously [21]. LiPF_6_ was obtained from Sigma-Aldrich (Saint Luis, MO, USA) (battery grade). Ethylene carbonate (EC) and dimethyl carbonate (DMC) were obtained from BASF (Ludwigshafen am Rhein, Germany) (battery grade).

The initial concentration range of an additive was based on the typical battery industry additives concentrations (fluoroethylene carbonate (FEC), vinylene carbonate (VC), etc.). Basic solvent mixture and lithium salt concentrations were based on our previous results [32], backed by Berhaut et al.’s results [58] in the case of LiTDI and by Ding et al.’s results [59] for LiPF_6_. Optimal concentrations in terms of cation conductivity were chosen for this work. The following electrolytes were employed as a result: the in-house 0.63 mol·kg^−1^ LiTDI in EC:DMC (1:2 weight ratio) and 1 mol·kg^−1^ LiPF_6_ in EC:DMC (1:2 weight ratio) used for comparison as an industrial standard. Solvent mixture description (EC:DMC, 1:2 weight ratio) is generally omitted in descriptions as it is the one and only description used throughout the results in the present paper. The LiHDI additive was added to electrolytes. LiHDI was incorporated in a 1–10% additional weight range, which is a typical range for commercial additive content. Samples of electrolytes were prepared in an argon-filled Mbraun (Garching, Germany) glovebox with less than 1 ppm moisture content and less than 0.5 ppm oxygen content.

Ionic conductivity measurements were performed using electrochemical impedance spectroscopy (EIS) in temperatures ranging from 0 to 50 °C. Electrolyte samples were put into a micro conductivity cell with the cell’s constant values of 0.3–0.7 cm^−1^ calibrated with a precision of 0.3%. Cells were then placed in a cryostat–thermostat system (Haake (Vreden, Germany) K75 with a DC50 temperature controller). Impedance measurements were carried out on the computer-interfaced multichannel potentiostat with a frequency response analyser option Bio-Logic Science Instruments (Seyssinet-Pariset, France) VMP3 within the 500 kHz–100 mHz frequency range with 10 points per decade and 5 mV A.C. signal amplitude. Samples for conductivity measurements were prepared in an argon-filled glovebox with less than 1 ppm moisture content and less than 0.5 ppm oxygen content.

Cyclic voltammetry was performed for samples containing 0.63 mol·kg^−1^ LiTDI electrolytes without additives with 2% LiHDI, as well as with both 10% FEC and 2% VC. Measurements were made in a two-electrode system with graphite (MTI-XTL) as a working electrode and metallic lithium (1.5 mm thick, 99.99%, Aldrich, Saint Luis, MO, USA) as counter and reference electrodes. Measurement started at 2 V vs. Li and was cycled between 0 and 1.5 V vs. Li with a 1 mV/s rate. Measurements were carried out at 25 °C.

Galvanostatic cycling was performed for full cells containing the graphite anode (MTI-XTL, Richmond, CA, USA, 2.6 mAh/cm^2^; 0.015 mm thick copper foil; 88 mg/cm^3^) and LiFePO_4_ cathode (MTI-XTL, 1.3 mAh/cm^2^; 0.015 mm thick aluminium foil; 100 mg/cm^3^) separated with a polypropylene separator (Celgard, 25 μm thickness). Cycling was performed in coin cells. The following electrolytes were prepared for cycling.

- 0.63 mol·kg^−1^ LiTDI in 1:2 weight ratio EC/DMC mixture;
- 0.63 mol·kg^−1^ LiTDI in 1:2 weight ratio EC/DMC mixture with 2% of LiHDI additive;
- 0.63 mol·kg^−1^ LiTDI in 1:2 weight ratio EC/DMC mixture with 3% of LiHDI additive;
- 0.63 mol·kg^−1^ LiTDI in 1:2 weight ratio EC/DMC mixture with 2% of VC and 10% of FEC additives;
- 1 mol·kg^−1^ LiPF_6_ in 1:2 weight ratio EC/DMC mixture with 2% of VC and 10% of FEC additives.

Cycling at 25 °C was performed for all the aforementioned electrolytes, while cycling at 60 °C was performed only for LiTDI electrolytes containing 2% of LiHDI, FEC/VC additives, and no additives. During cycling, samples were stored in the oven (Memmert, Schwabach, Germany). Samples prepared for cycling at 60 °C were initially cycled at 25 °C during formation and the 5 subsequent cycles. Subsequently, samples were stored at 60 °C for 48 h for thermostating, which was followed by actual cycling. Cycling at 25 °C was performed twice for every electrolyte. The first cycling was performed with both electrodes of the same size (17 mm diameter; 5.9 mAh anode’s capacity; 3.0 mAh cathode’s capacity); thus, LiFePO_4_ (cathode) was a capacity-limiting electrode. The second cycling was performed with smaller anodes (11 mm diameter; 2.5 mAh anode’s capacity), during which graphite (anode) was a capacity-limiting electrode. Cells with larger electrodes were charged, discharged, and charged again with 100 µA current and then cycled with 500 µA current. The current for smaller electrodes was smaller, respectively; for formation cycles, it was 42 µA, and for cycling, it was 220 µA. Cycling was performed with a potential limitation of 2.1–3.6 V. After each half-cycle, there was a 2 h-long intermission.

Impedance spectra for cells cycled at 25 °C were measured during cycling. Impedance spectra were taken at the charged state at the end of each intermission. Galvanostatic cycling and impedance measurements were carried out on the computer-interfaced multichannel potentiostat with a frequency response analyser option Bio-Logic Science Instruments VMP3. The spectra were fitted to an equivalent circuit (presented in Figure S1) using an equivalent-circuit 4.55 application developed by B.A. Boukamp [60].

Electrode surfaces of the graphite anode and LiFePO_4_ cathode were studied via X-ray photoelectron spectroscopy (XPS). Depth profiles of samples after formation cycles and after 50 cycles were performed for the electrodes’ samples cycled with the following electrolytes: 0.63 mol·kg^−1^ LiTDI without additives, 0.63 mol·kg^−1^ LiTDI with 2% LiHDI additive, and 0.63 mol·kg^−1^ LiTDI with 10% FEC and 2% VC additives. In order to prepare electrodes for XPS experiments, cells cycled up to the required number of cycles have been disassembled in the argon-filled drybox. The electrodes were taken out then rinsed with DMC and dried in a vacuum oven (VO 400, Memmert) at 40 °C with slowly decreasing pressure.

The XPS measurements were carried out on an Escalab 250Xi multispectroscope (Thermo Fisher Scientific, Waltham, MA, USA). In order to determine the surface chemistry of the analysed samples, the high-resolution spectra were recorded in the binding energy range of F1s, Li1s, O1s, N1s, and C1s peaks. The utilised spectroscope is equipped with a monochromatic Al Kα energy source. The applied X-ray spot diameter was 250 µm. The pass energy for the photoelectrons through the hemisphere analyser was set to 20 eV, and the energy step size was 0.1 eV. Prior to the operation, the spectroscope was calibrated on Cu and Au single crystals. The charge compensation of the investigated samples was controlled through low-energy electron and Ar^+^ ion flow by means of a flood gun. Finally, due to the presence of surface ionisation effects, the XPS spectra were calibrated to C1s graphite at 284.0 eV. No other form of sample pre-treatment was performed; thus, the air exposure of the investigated samples was minimised. Spectral deconvolution was conducted with Avantage v5.973 software (Thermo Fisher Scientific).

Thermal analysis was performed using differential scanning calorimetry (DSC). The following samples were prepared:

- Graphite;
- LiFePO_4_;
- 0.63 mol·kg^−1^ LiTDI in EC:2DMC with 2% of LiHDI;
- 0.63 mol·kg^−1^ LiTDI in EC:2DMC with 2% of VC and 10% of FEC;
- 0.63 mol·kg^−1^ LiTDI in EC:2DMC with 2% of LiHDI + graphite;
- 0.63 mol·kg^−1^ LiTDI in EC:2DMC with 2% of VC and 10% of FEC + graphite;
- 0.63 mol·kg^−1^ LiTDI in EC:2DMC with 2% of LiHDI + LiFePO_4_;
- 0.63 mol·kg^−1^ LiTDI in EC:2DMC with 2% of VC and 10% of FEC + LiFePO_4_;
- 0.63 mol·kg^−1^ LiTDI in EC:2DMC with 2% of LiHDI + graphite (lithiated);
- 0.63 mol·kg^−1^ LiTDI in EC:2DMC with 2% of VC and 10% of FEC + graphite (lithiated).

Samples were measured in aluminium hermetic pans during heating from 0 °C to 100 °C with a scanning rate of 10 °C/min. A Q200 calorimeter (TA Instruments, New Castle, DE, USA) was used.

## 4. Conclusions

To summarise, the application of LiHDI as an SEI layer-stabilising additive for lithium electrolytes was tested. The addition of LiHDI to a LiTDI-based electrolyte allowed us to obtain higher conductivity than with an electrolyte containing FEC and VC. This shows that LiHDI does not negatively impact electrolyte performance. Cyclic voltammetry showed that SEI layer formation with LiHDI occurs at higher potentials than SEI formation with LiTDI. The impact of LiHDI additive on cycling and SEI stability was investigated with galvanostatic cycling, impedance study, and XPS measurements. It was found that 2% of LiHDI provides the best results. Capacity retention was tested for the graphite-LiFePO_4_ system. By testing systems with both an anode and a cathode as capacity-limiting electrodes, it was shown that LiHDI additive improves capacity retention. The additive provided better results for the system with the cathode as the capacity-limiting electrode. This shows that the capacity loss due to the lithium side reactions is less problematic for samples with LiHDI, while it is not as good as FEC and VC in the prevention of anode and SEI degradation. It was also found that the addition of FEC and VC results in the selective blocking of the first stage of intercalation for graphite anodes. The XPS results revealed that the contents of oxygen increased, and the contents of fluorine decreased during cycling with FEC/VC. For samples with LiHDI, the reverse is true. This might suggest that a higher content of oxygen and a lower content of fluorine are beneficial for SEI stability.

To sum up, LiHDI does not provide as good a capacity retention as FEC and VC. However, the situation is diametrically different at elevated temperatures. Above 60 °C, LiHDI retains good properties, while samples with FEC and VC quickly lose capacity.

## Figures and Tables

**Figure 7:**
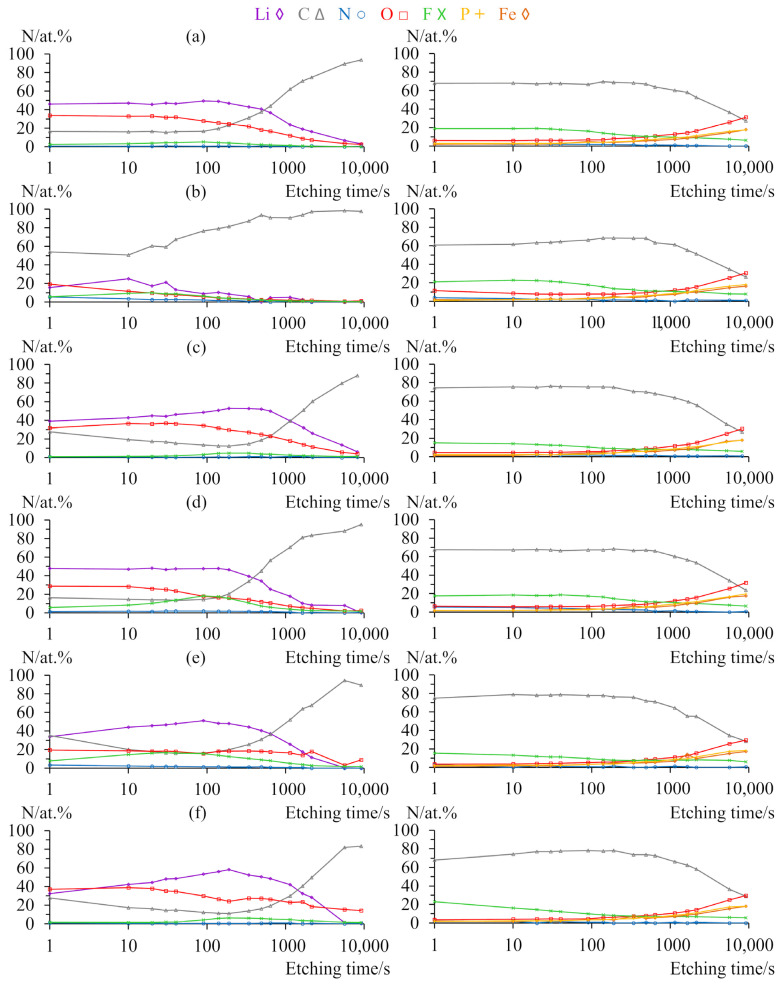
XPS depth profiles of electrodes showing relative contents of selected elements. Anode profiles are on the left and cathode profiles are on the right. (**a**) LiTDI without additives after formation. (**b**) LiTDI without additives after 50 cycles. (**c**) LiTDI with 2% of LiHDI after formation. (**d**) LiTDI with 2% of LiHDI after 50 cycles. (**e**) LiTDI with FEC and VC after formation. (**f**) LiTDI with FEC and VC after 50 cycles.

## Data Availability

The data presented in this study are available in article and Supplementary Materials.

## Supplementary Materials

The following supporting information can be downloaded at https://www.mdpi.com/article/10.3390/molecules29040804/s1. Exemplary impedance spectrum and equivalent circuit scheme (Figure S1); Structural formula of LiTDI (Formula S1); Structural formula of LiHDI (Formula S2); Thermograms of DSC measurements during heating with 10 °C/min. (Figure S2); Assignment of the XPS signals to the compounds for anodes and cathodes (Table S1); Relative content of moieties/elements for untreated anode based on XPS measurement (Table S2); Relative content of moieties/elements for untreated cathode based on XPS measurement (Table S3); Depth profiles of anodes showing the chemical environment of lithium, fluorine, oxygen, and nitrogen for the samples after formation and after 50 cycles with LiTDI, LiTDI—2%LiHDI, and LiTDI—FEC/VC electrolytes (Figures S3–S26); Depth profiles of cathodes showing the chemical environment of fluorine, oxygen, and nitrogen for the samples after formation and after 50 cycles with LiTDI, LiTDI—2%LiHDI, and LiTDI—FEC/VC electrolytes (Figures S27–S44); Pictures of two electrolytes: 1–0.63 M LiTDI EC:2DMC + 2% LiHDI, 2–0.63 M LiTDI EC:2DMC + 2% VC + 10% FEC, (a) Freshly prepared (b) Stored for 48 h at 60℃ (Scheme S1); Resistances of (a) electrolyte, (b) interface of Li|electrolyte|Li cells with 0.63 M LiTDI EC:2DMC electrolytes with various amounts of LiHDI additive (Figure S45); Fitting parameters for impedance measurements of cell without additives at 25 °C (Table S4); Fitting parameters for impedance measurements of the cell with FEC-VC at 25 °C (Table S5); Fitting parameters for impedance measurements of the cell with LiHDI at 25 °C (Table S6); Fitting parameters for impedance measurements of the cells at 60 °C (Table S7).
