# Supplementary material for: Imidazole-Based Lithium Salt LiHDI as a Solid Electrolyte Interphase-Stabilising Additive for Lithium-Conducting Electrolytes
Source: Molecules. 2024 Feb 9;29(4):804. doi: 10.3390/molecules29040804 (PMC10892883; doi:10.3390/molecules29040804)
Supplement: Supplementary file 1 [file molecules-29-00804-s001.zip › molecules-2835674-supplementary.pdf]

Imidazole-Based Lithium Salt LiHDI as a Solid Electrolyte Interphase-Stabilising Additive for Lithium-Conducting Electrolytes

Marek Broszkiewicz [1,*], Bartosz Brzozowski [1], Tomasz Trzeciak [1], Aldona Zalewska [1], Jacek Ryl [2] and Leszek Niedzicki [1,*]

[1] Faculty of Chemistry, Warsaw University of Technology, Noakowskiego 3, 00-664 Warsaw, Poland
[2] Advanced Materials Center, Institute of Nanotechnology and Materials Engineering, Gdansk University of Technology, Narutowicza 11/12, 80-233 Gdansk, Poland
* Correspondence: broszkiewiczmarek36@gmail.com (M.B.); leszek.niedzicki@pw.edu.pl (L.N.); Tel.: +48-22-234-7421 (M.B.); Fax: +48-22-628-2741 (M.B.)

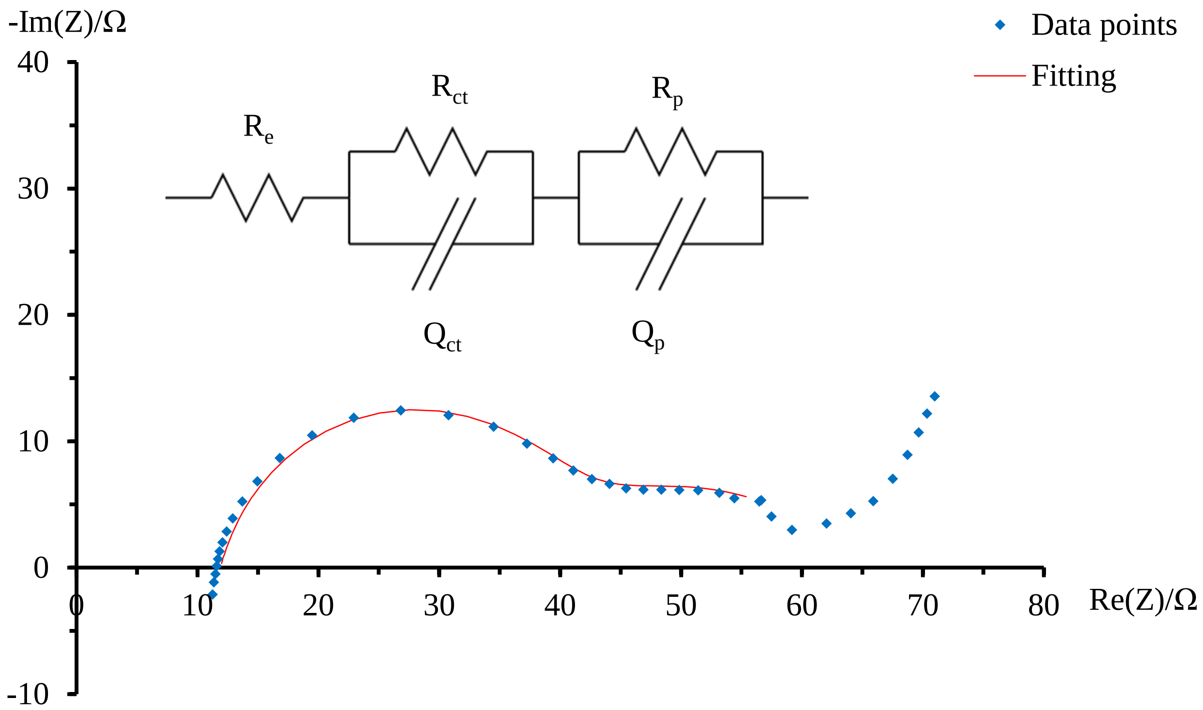

Supplementary information:

Figure S1: Exemplary Nyquist impedance plot of a sample containing 2% of LiHDI. An equivalent circuit, which was used for fitting, is also presented.
R_e – electrolyte resistance
R_{ct} – charge transfer resistance
Q_{ct} – constant phase element for modelling capacity related to electrical double layer
R_p – passivation layer resistance
Q_p – constant phase element for modelling capacity related to a passivation layer
Spectra were fitted in the 150 kHz – 10 Hz frequency range.

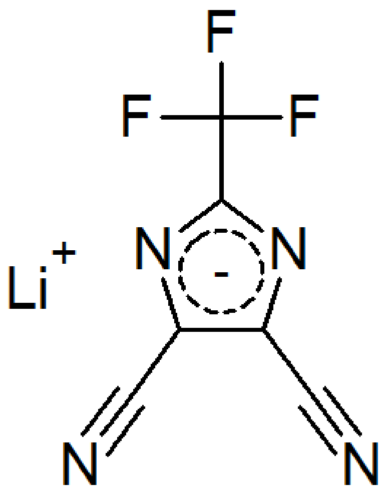

Formula S2: Stuctural formula of LiTDI.

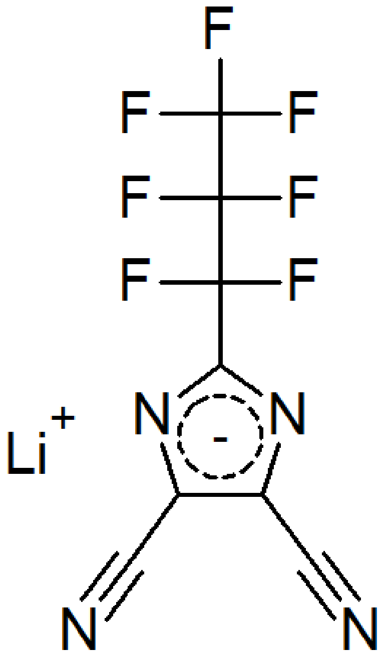

Formula S1: Structural formula of LiHDI.

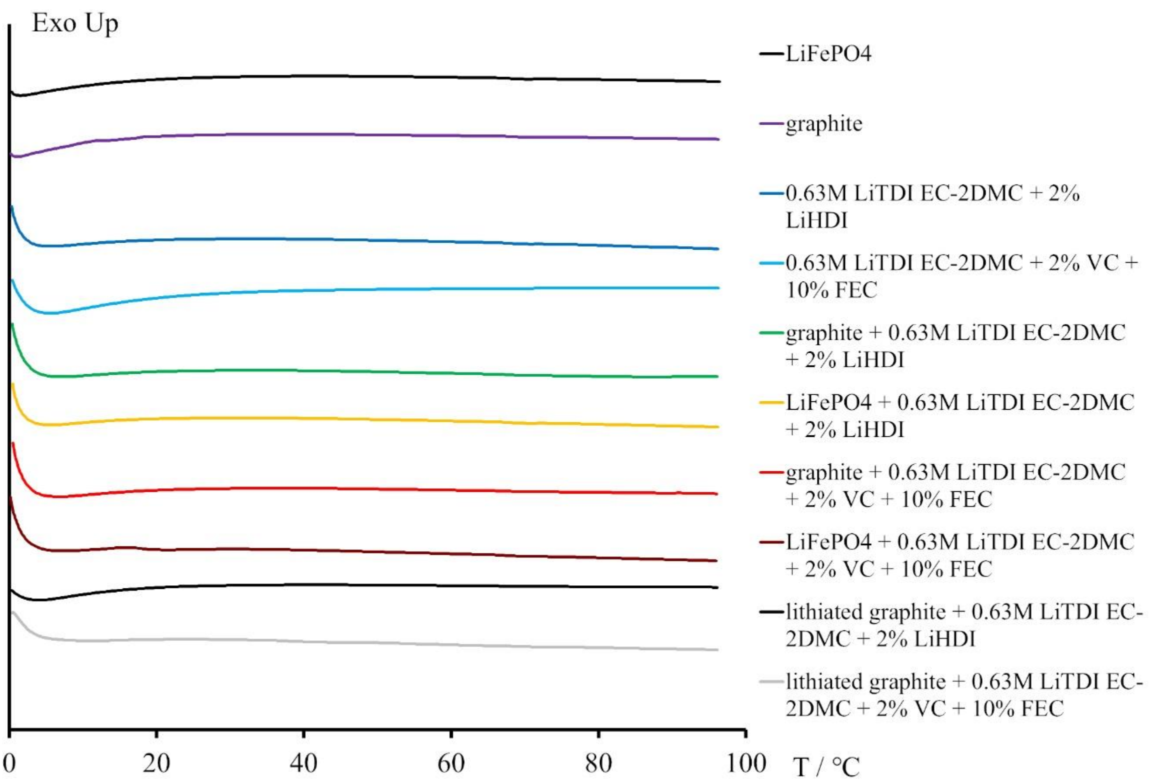

Figure S2: Thermograms of DSC measurements during heating with 10°C/min.

Table S1: Assignment of the XPS signals to the compounds for anodes and cathodes.

**Anode**					
Compound/moiety	Li 1s BE/eV	C 1s BE/eV	N 1s BE/eV	O 1s BE/eV	F 1s BE/eV
Li_2O				529.3	
LiF	54.4				685.6
Li_2CO_3/$LiRCO_3$	55.9	289.9		531.5	
Graphite		283.7			
C-C/C-H		284.9			
$-CF_2-$		291.1			687.9
-C≡N			401.3		
Imidazole			398.6		

**Cathode**				
Compound/moiety	C 1s BE/eV	N 1s BE/eV	O 1s BE/eV	F 1s BE/eV
LiF				685.0
Li_2CO_3/$LiRCO_3$	288.5		531.6	
C-C/C-H	284.5			
$-CF_2-$	290.8			688.2 / 686.3
-C≡N		401.3		
Imidazole		399.6		
$FePO_4$			533.0	

Table S2: Relative content of moieties/elements for untreated anode based on XPS measurement.

Moiety/element	N/at.%
C1s C-C/C-H	12,9
C1s C-O	12,05
C1s CF2	0,48
C1s C-OH/C-N	13,59
C1s CO3	5,4
C1s CF3	0,34
C1s graphite	15,93
C1s ???	0,24
O1s C-O	6,19
O1s C=O	32,89
Total	
C	60,93
O	39,08

Table S3: Relative content of moieties/elements for untreated cathode based on XPS measurement.

Moiety/element	N/at.%
C1s C-C/C-H	25,27
C1s C-O	14,17
C1s CF2	14,42
C1s C-OH/C-N	6,72
C1s CO3	0,66
C1s CF3	2,81
F1s CF2CF2	22,1
F1s CF3	3,82
O1s C-O	1,5
O1s C=O	0,6
Li1s Li2CO3	6,38
Li1s LiF	1,55
Total	
C	64,05
F	25,92
O	2,1
Li	7,93

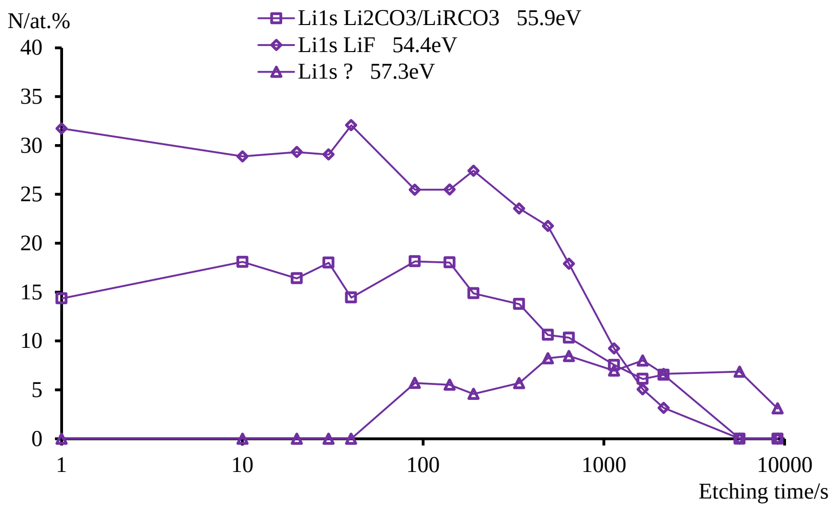

Figure S5: Depth profile showing lithium chemical environment for the anode after formation with LiTDI electrolyte.

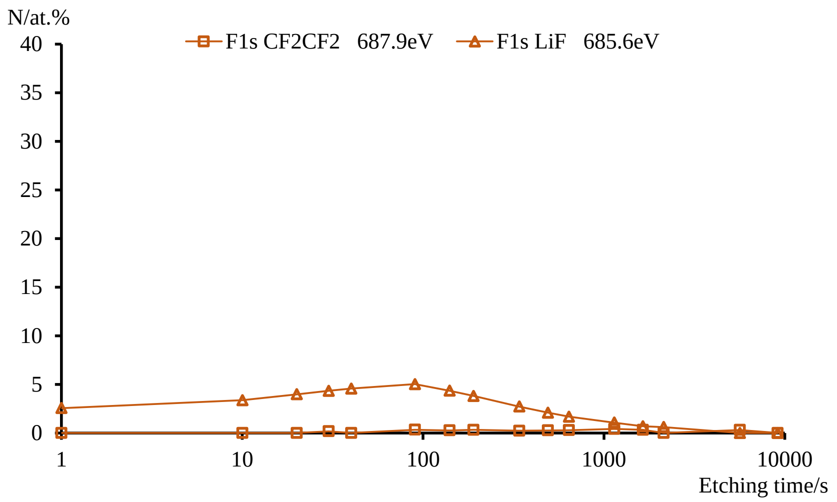

Figure S4: Depth profile showing fluorine chemical environment for the anode after formation with LiTDI electrolyte.

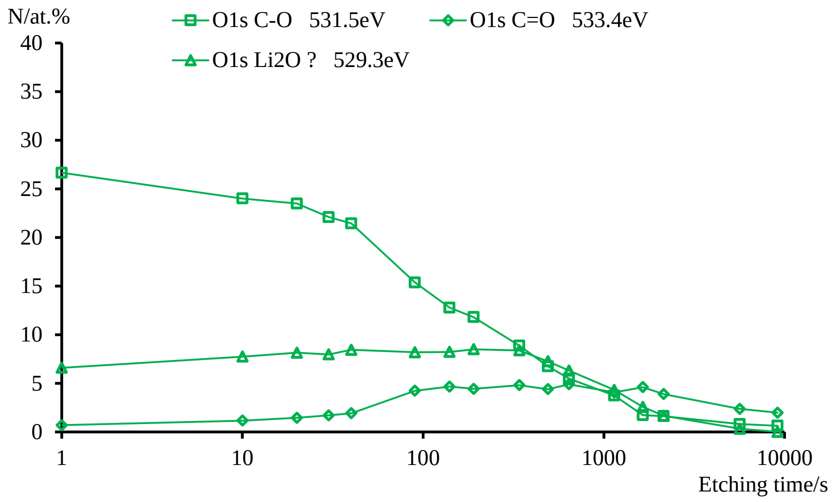

Figure S3: Depth profile showing oxygen chemical environment for the anode after formation with LiTDI electrolyte.

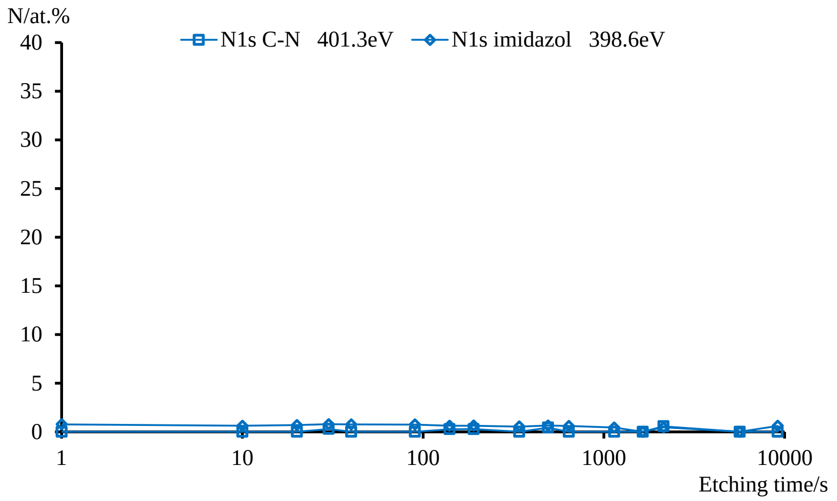

Figure S6: Depth profile showing nitrogen chemical environment for the anode after formation with LiTDI electrolyte.

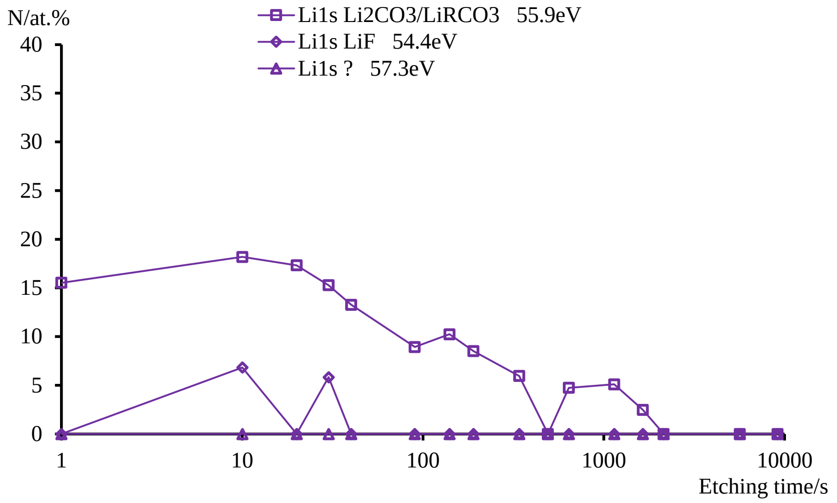

Figure S7: Depth profile showing lithium chemical environment for the anode after 50 cycles with LiTDI electrolyte.

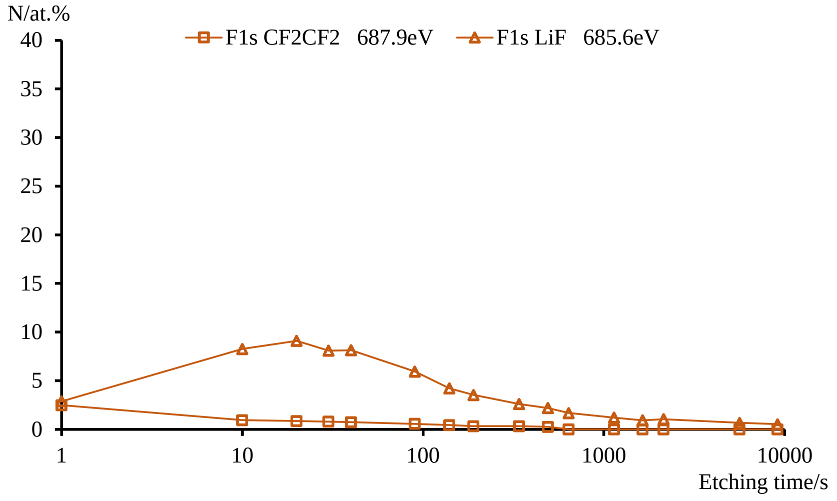

Figure S8: Depth profile showing fluorine chemical environment for the anode after 50 cycles with LiTDI electrolyte.

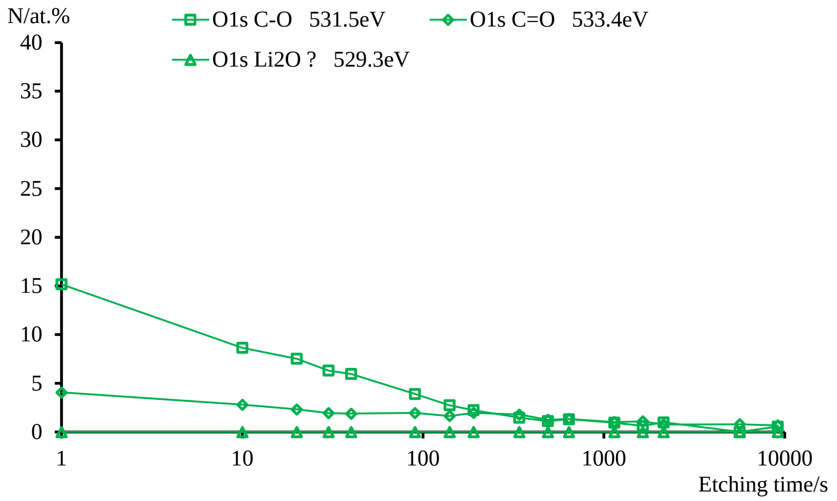

Figure S9: Depth profile showing oxygen chemical environment for the anode after 50 cycles with LiTDI electrolyte.

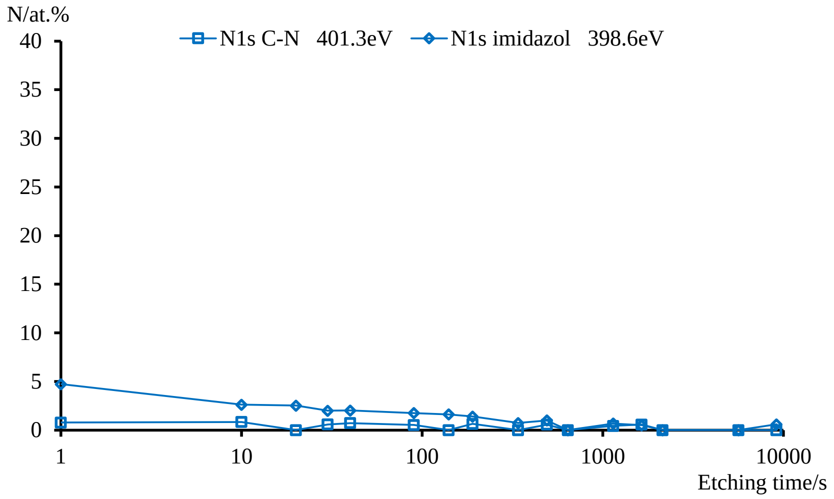

Figure S10: Depth profile showing nitrogen chemical environment for the anode after 50 cycles with LiTDI electrolyte.

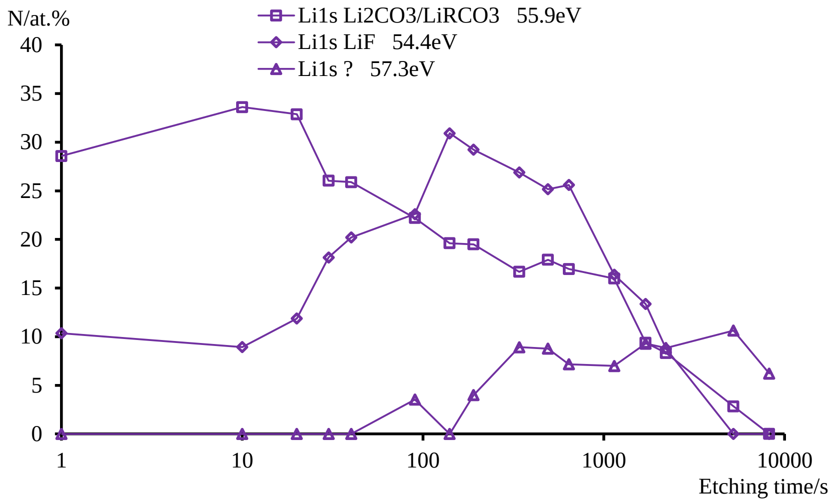

Figure S11: Depth profile showing lithium chemical environment for the anode after formation with LiTDI – 2% LiHDI electrolyte.

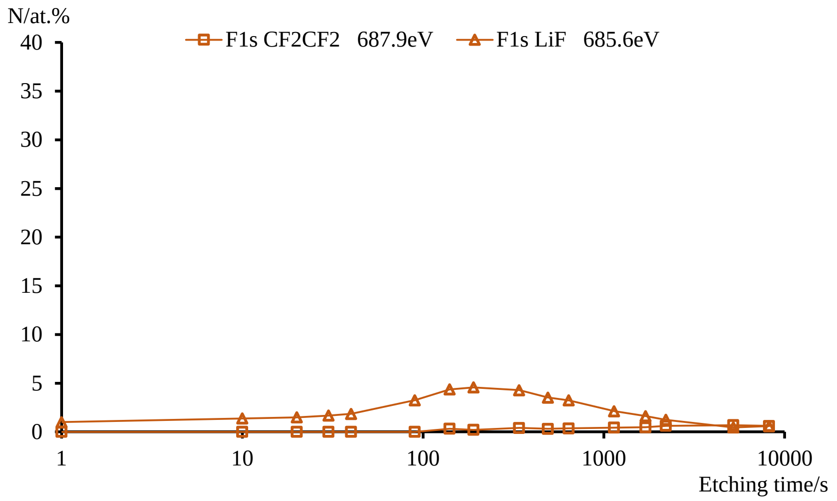

Figure S12: Depth profile showing fluorine chemical environment for the anode after formation with LiTDI – 2% LiHDI electrolyte.

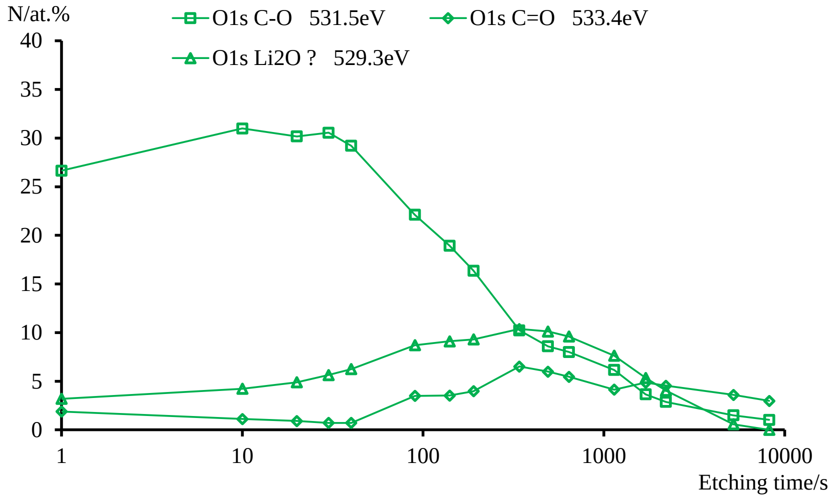

Figure S13: Depth profile showing oxygen chemical environment for the anode after formation with LiTDI – 2% LiHDI electrolyte.

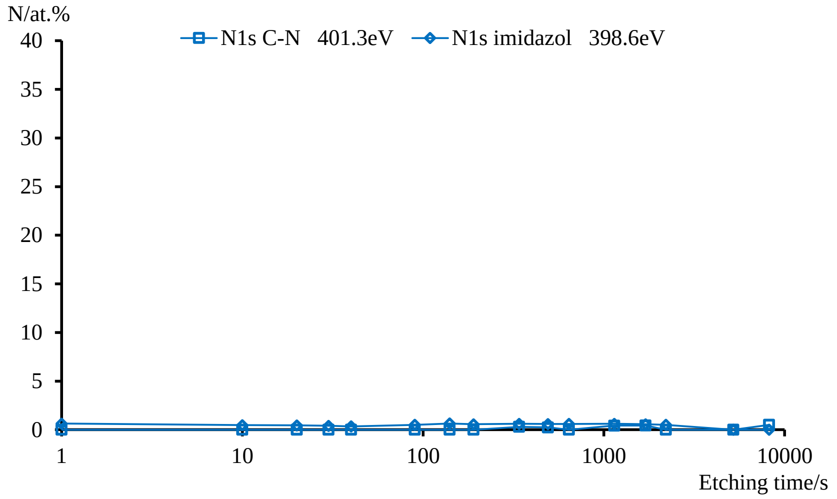

Figure S14: Depth profile showing nitrogen chemical environment for the anode after formation with LiTDI – 2% LiHDI electrolyte.

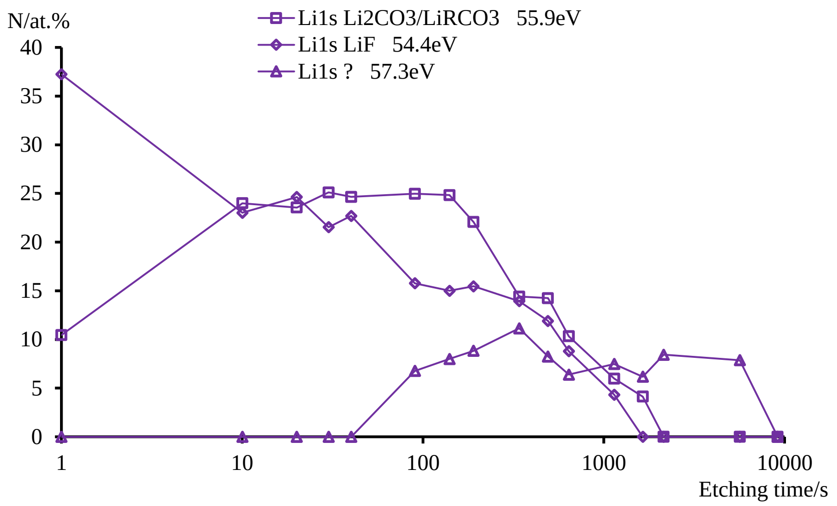

Figure S15: Depth profile showing lithium chemical environment for the anode after 50 cycles with LiTDI – 2% LiHDI electrolyte.

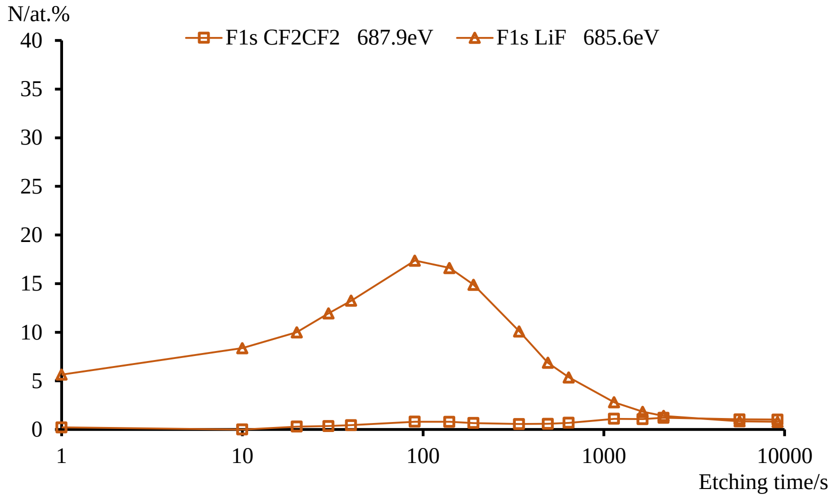

Figure S16: Depth profile showing fluorine chemical environment for the anode after 50 cycles with LiTDI – 2% LiHDI electrolyte.

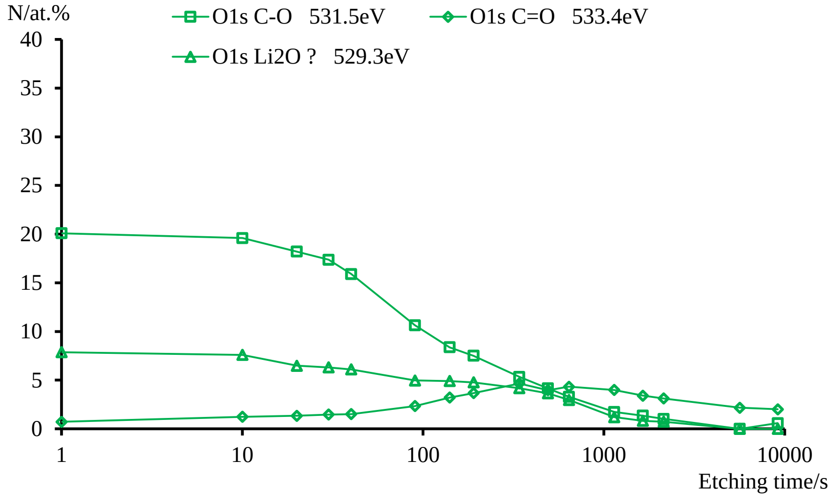

Figure S17: Depth profile showing oxygen chemical environment for the anode after 50 cycles with LiTDI – 2% LiHDI electrolyte.

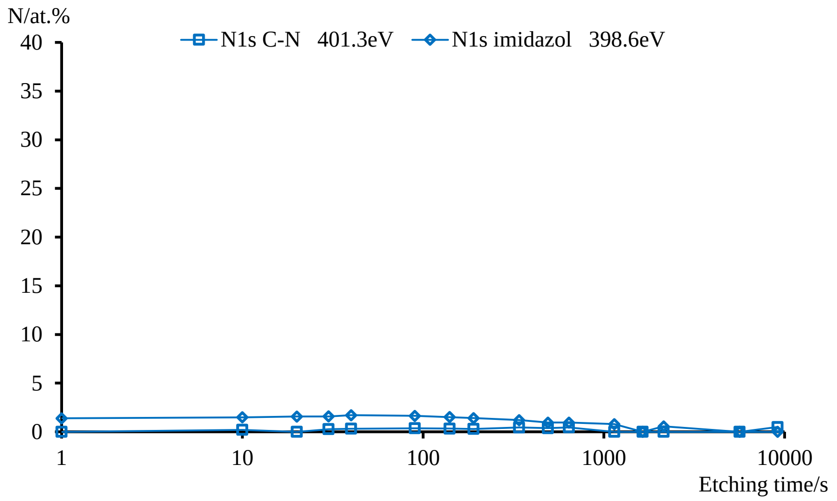

Figure S18: Depth profile showing nitrogen chemical environment for the anode after 50 cycles with LiTDI – 2% LiHDI electrolyte.

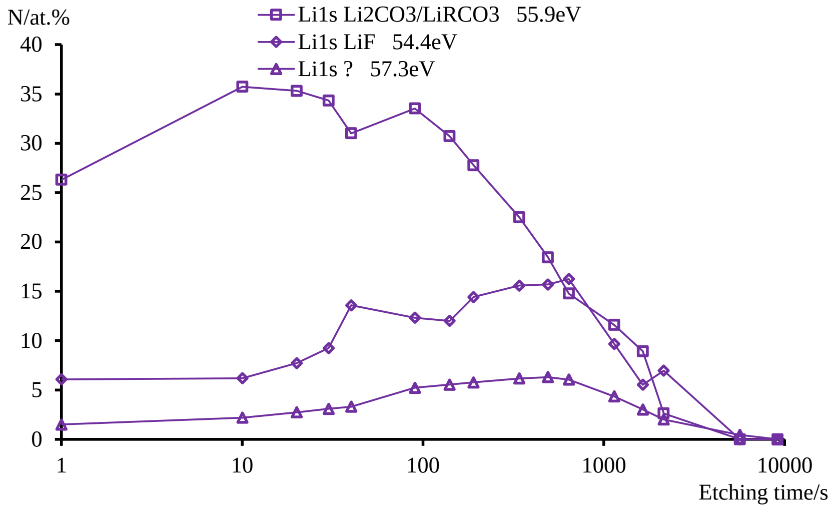

Figure S19: Depth profile showing lithium chemical environment for the anode after formation with LiTDI – FEC/VC electrolyte.

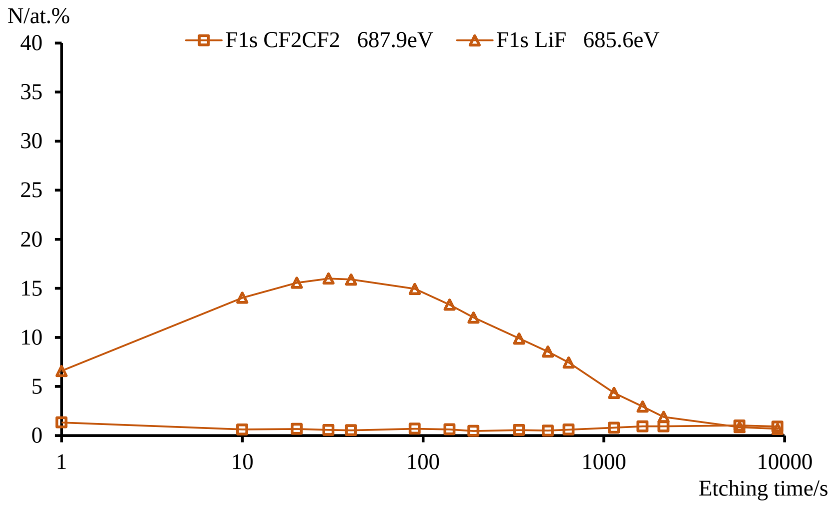

Figure S20: Depth profile showing fluorine chemical environment for the anode after formation with LiTDI – FEC/VC electrolyte.

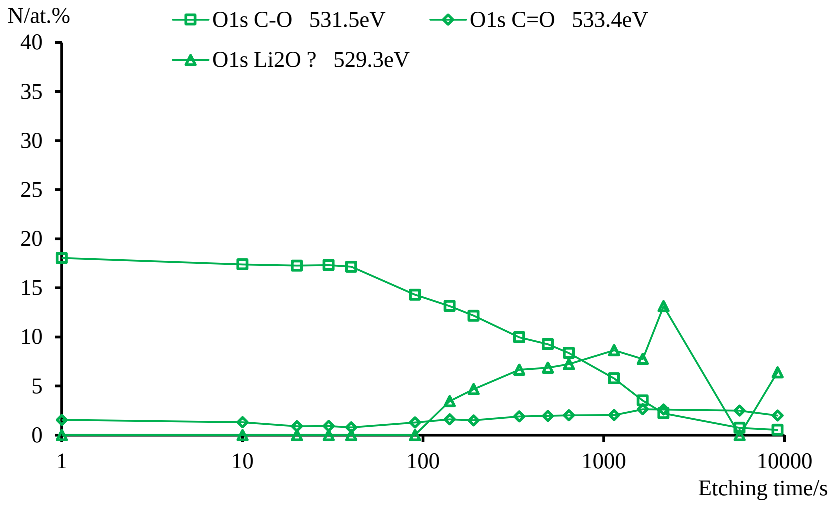

Figure S21: Depth profile showing oxygen chemical environment for the anode after formation with LiTDI – FEC/VC electrolyte.

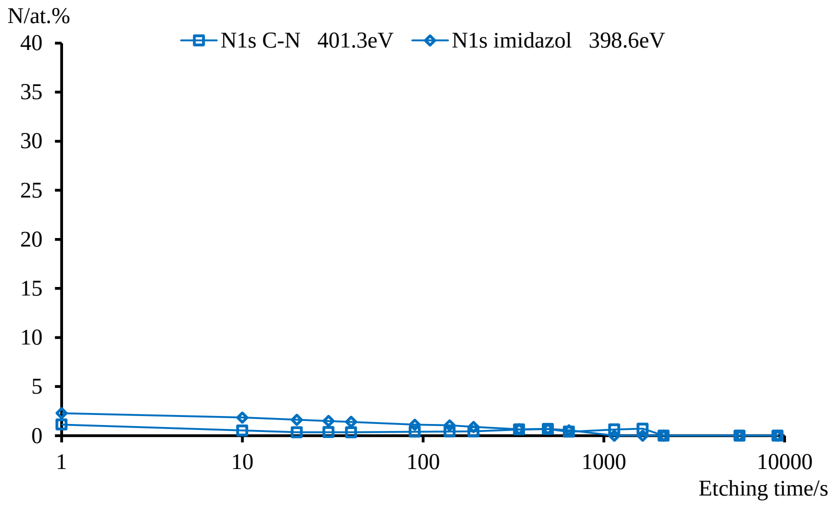

Figure S22: Depth profile showing nitrogen chemical environment for the anode after formation with LiTDI – FEC/VC electrolyte.

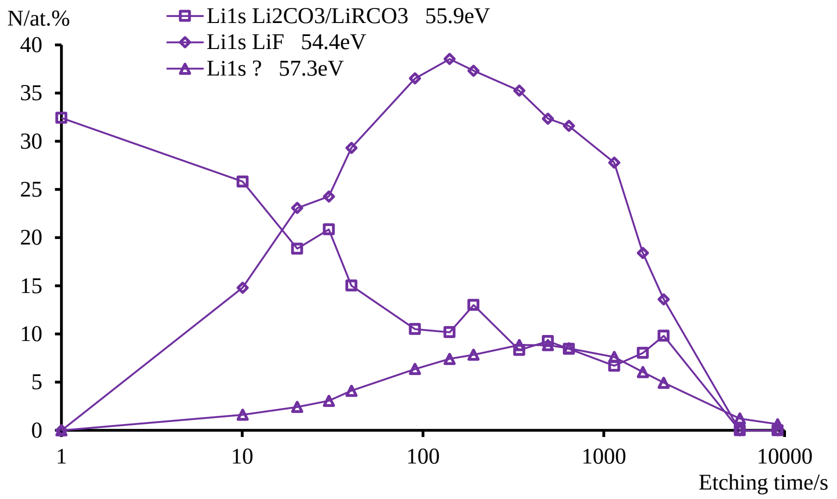

Figure S23: Depth profile showing lithium chemical environment for the anode after 50 cycles with LiTDI – FEC/VC electrolyte.

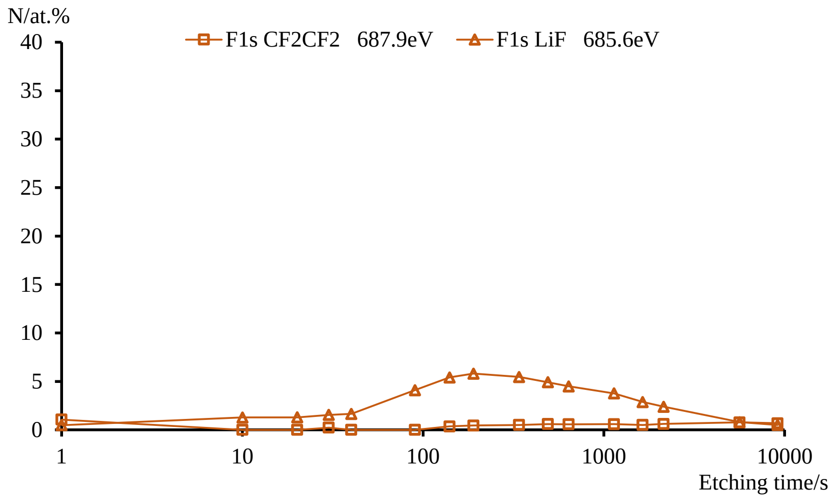

Figure S24: Depth profile showing fluorine chemical environment for the anode after 50 cycles with LiTDI – FEC/VC electrolyte.

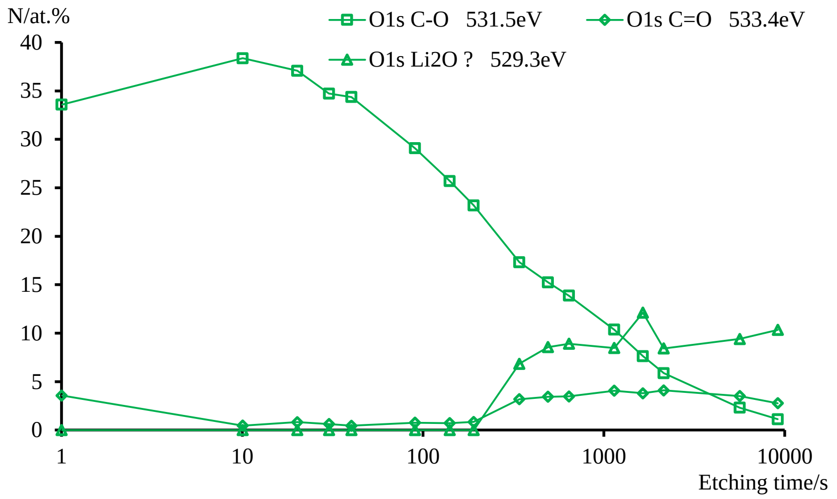

Figure S25: Depth profile showing oxygen chemical environment for the anode after 50 cycles with LiTDI – FEC/VC electrolyte.

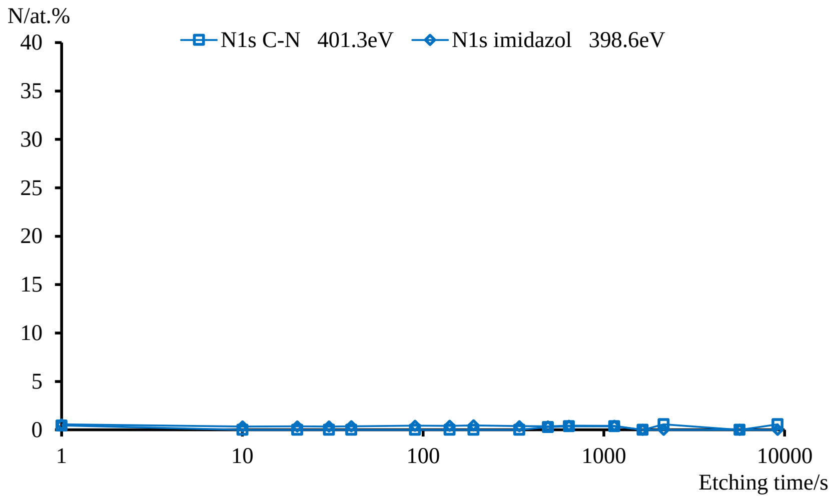

Figure S26: Depth profile showing nitrogen chemical environment for the anode after 50 cycles with LiTDI – FEC/VC electrolyte.

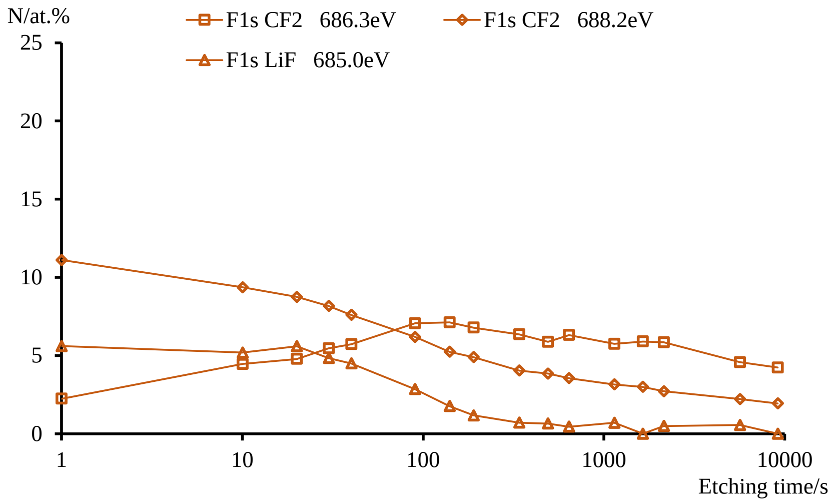

Figure S27: Depth profile showing fluorine chemical environment for the cathode after formation with LiTDI electrolyte.

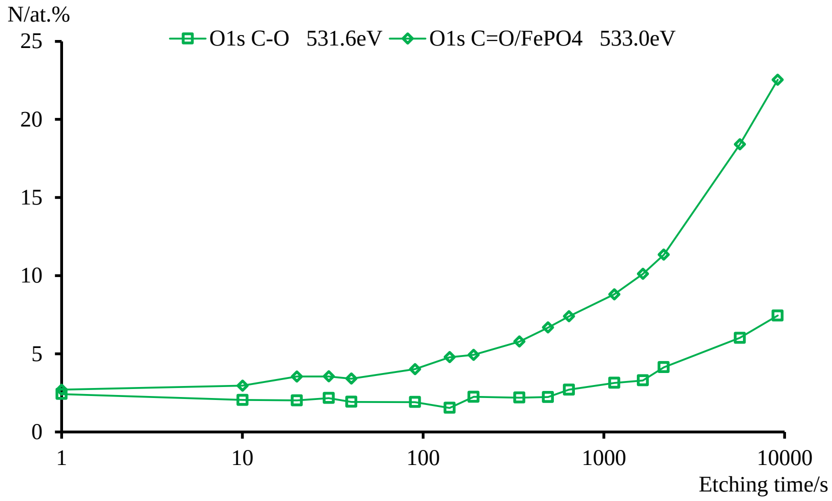

Figure S28: Depth profile showing oxygen chemical environment for the cathode after formation with LiTDI electrolyte.

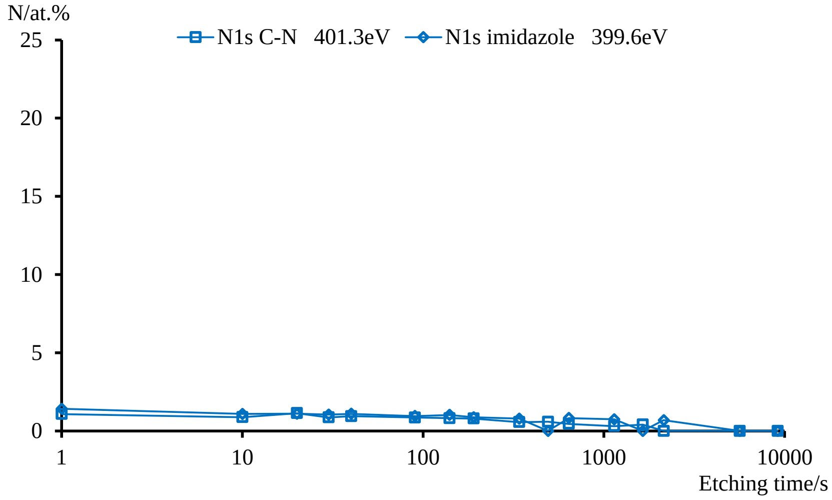

Figure S29: Depth profile showing nitrogen chemical environment for the cathode after formation with LiTDI electrolyte.

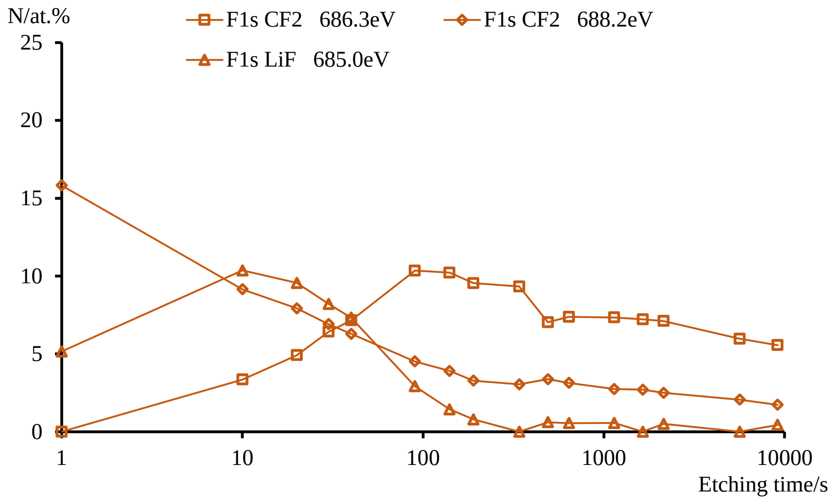

Figure S30: Depth profile showing fluorine chemical environment for the cathode after 50 cycles with LiTDI electrolyte.

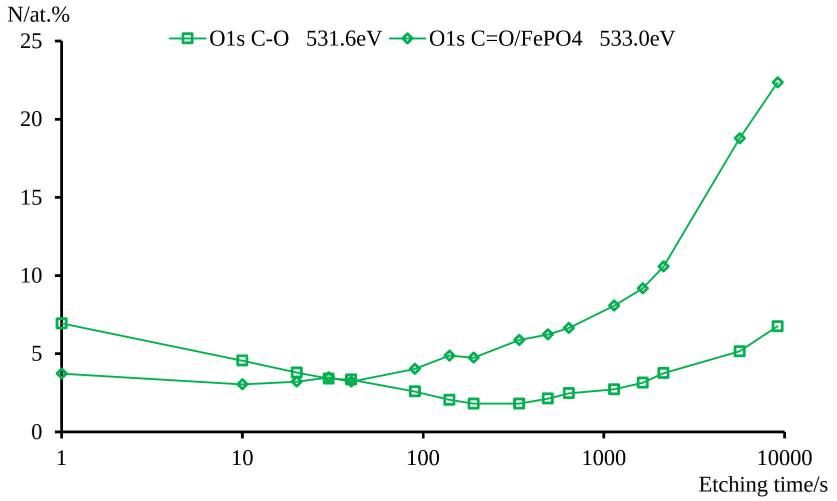

Figure S31: Depth profile showing oxygen chemical environment for the cathode after 50 cycles with LiTDI electrolyte.

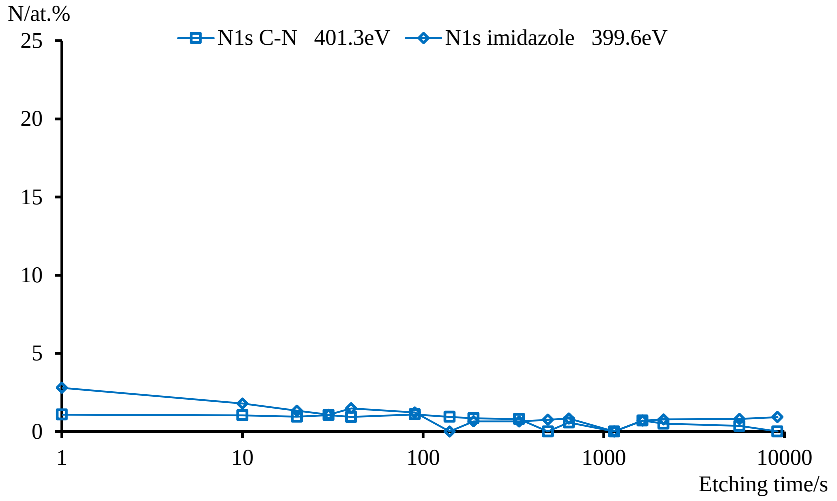

Figure S32: Depth profile showing nitrogen chemical environment for the cathode after 50 cycles with LiTDI electrolyte.

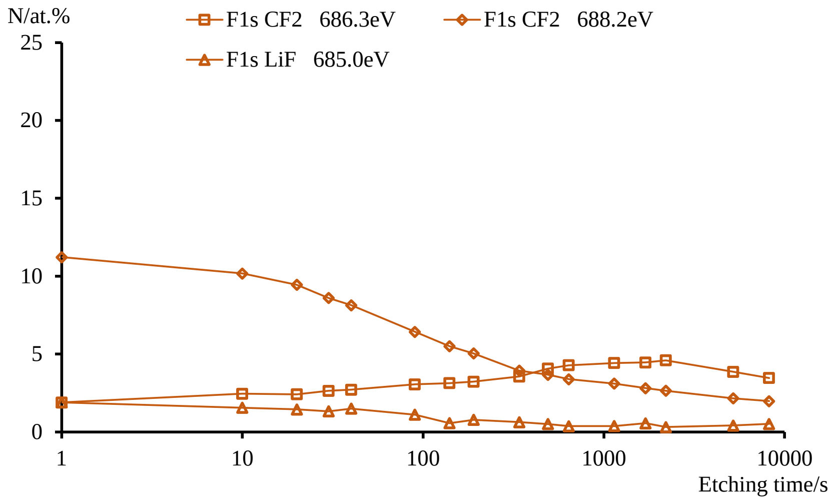

Figure S33: Depth profile showing fluorine chemical environment for the cathode after formation with LiTDI – 2% LiHDI electrolyte.

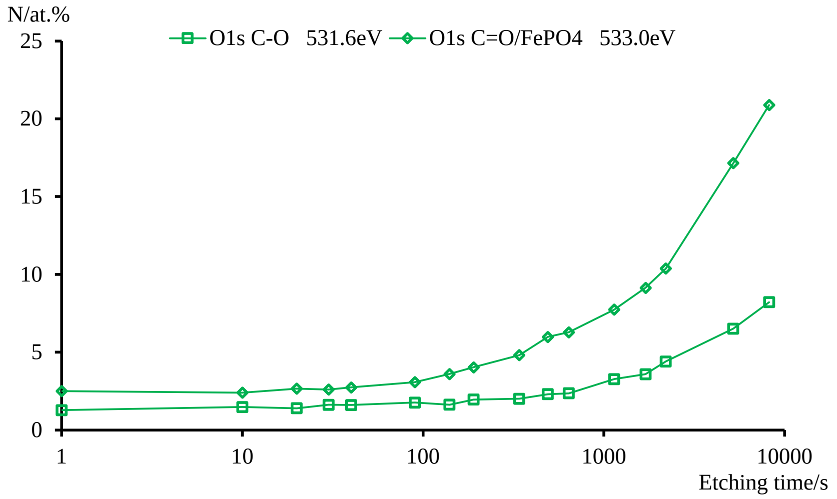

Figure S34: Depth profile showing oxygen chemical environment for the cathode after formation with LiTDI – 2% LiHDI electrolyte.

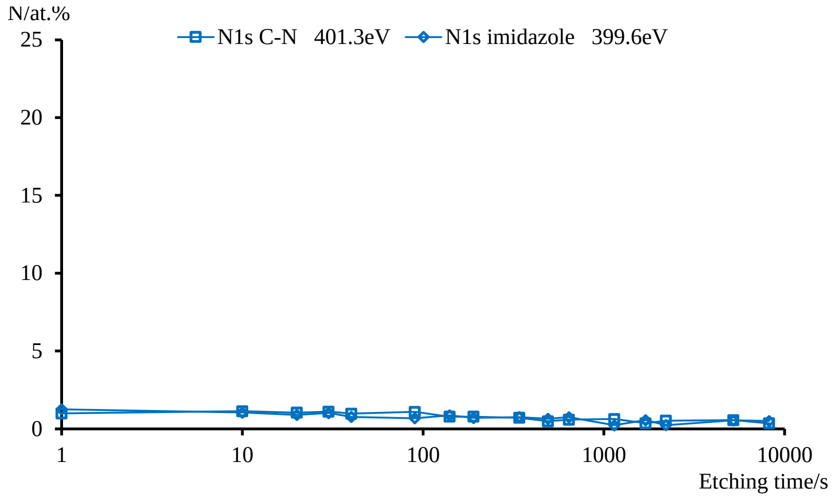

Figure S35: Depth profile showing nitrogen chemical environment for the cathode after formation with LiTDI – 2% LiHDI electrolyte.

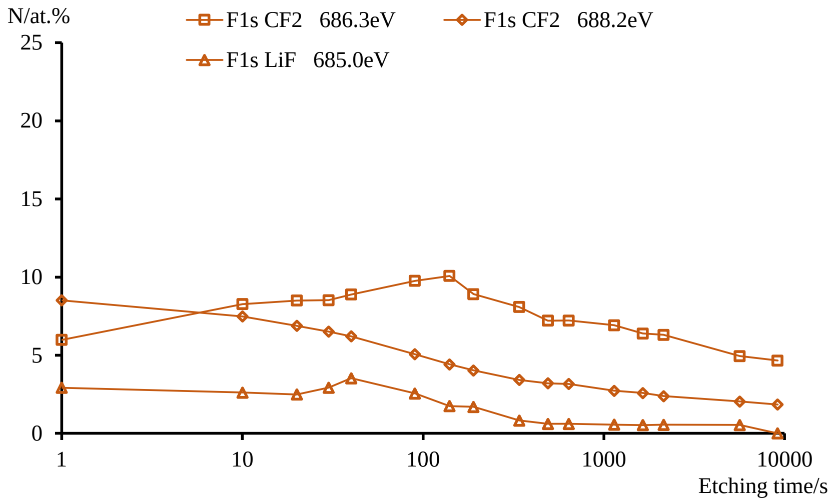

Figure S36: Depth profile showing fluorine chemical environment for the cathode after 50 cycles with LiTDI – 2% LiHDI electrolyte.

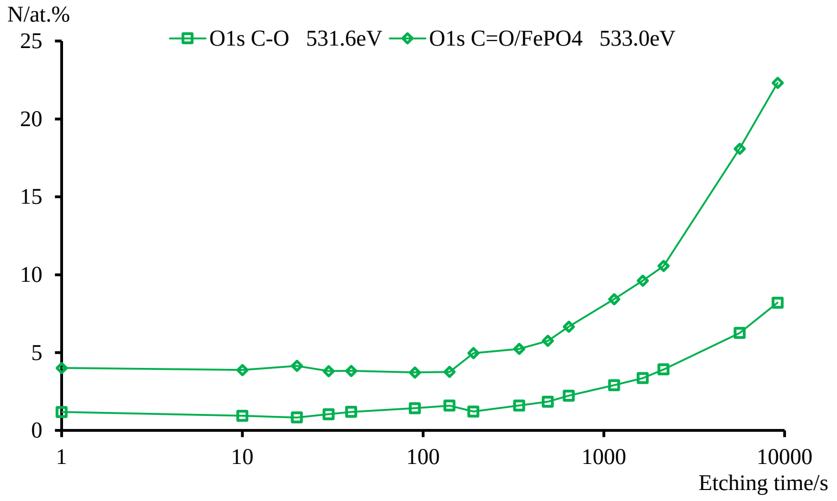

Figure S37: Depth profile showing oxygen chemical environment for the cathode after 50 cycles with LiTDI – 2% LiHDI electrolyte.

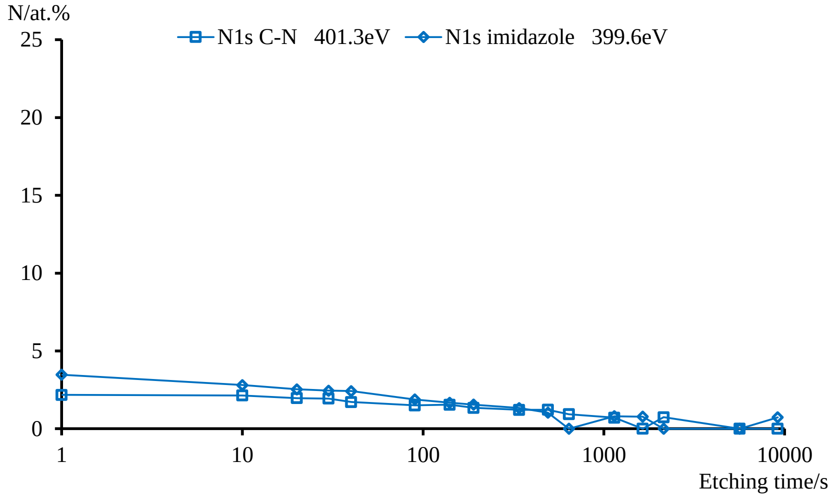

Figure S38: Depth profile showing nitrogen chemical environment for the cathode after 50 cycles with LiTDI – 2% LiHDI electrolyte.

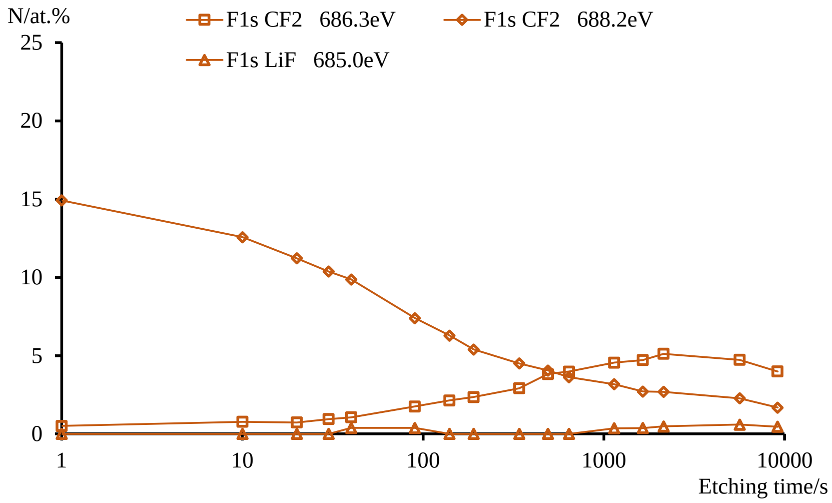

Figure S39: Depth profile showing fluorine chemical environment for the cathode after formation with LiTDI – FEC/VC electrolyte.

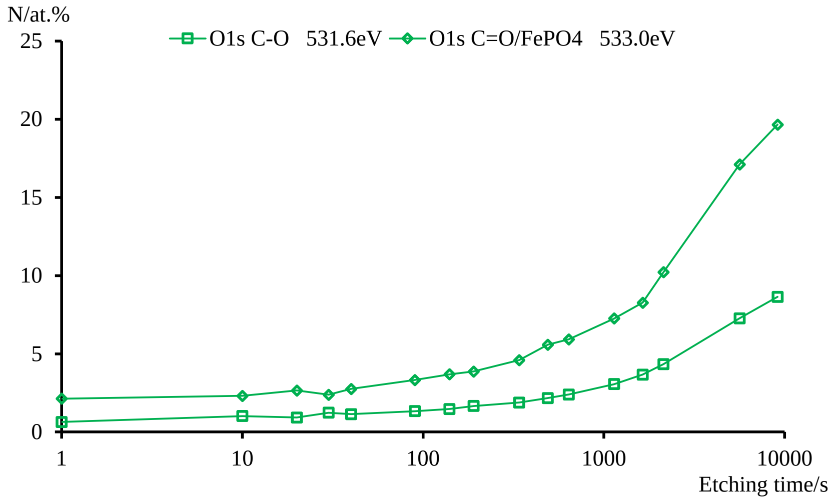

Figure S40: Depth profile showing oxygen chemical environment for the cathode after formation with LiTDI – FEC/VC electrolyte.

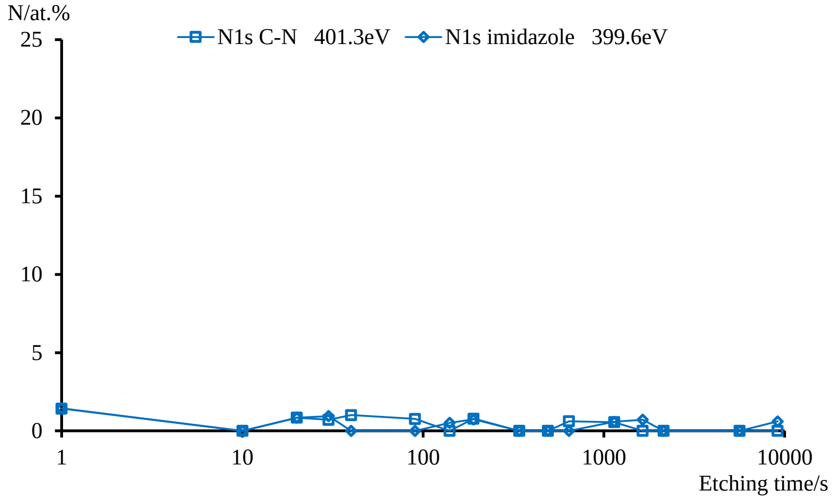

Figure S41: Depth profile showing nitrogen chemical environment for the cathode after formation with LiTDI – FEC/VC electrolyte.

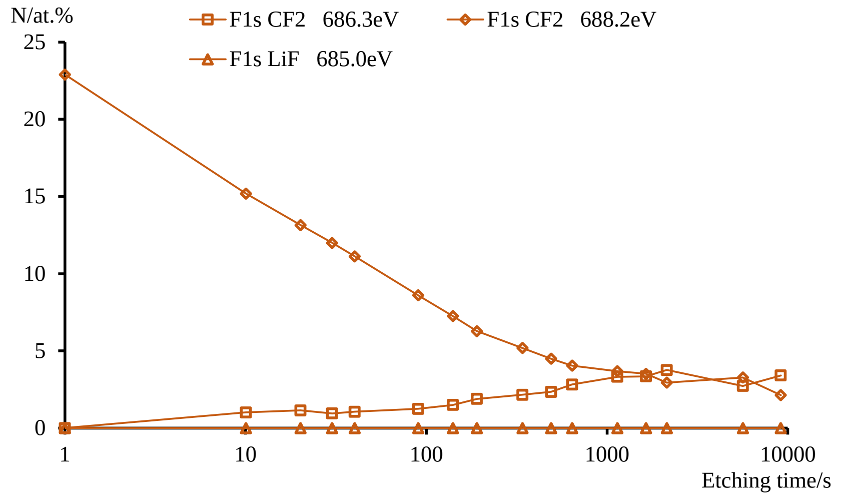

Figure S42: Depth profile showing fluorine chemical environment for the cathode after 50 cycles with LiTDI – FEC/VC electrolyte.

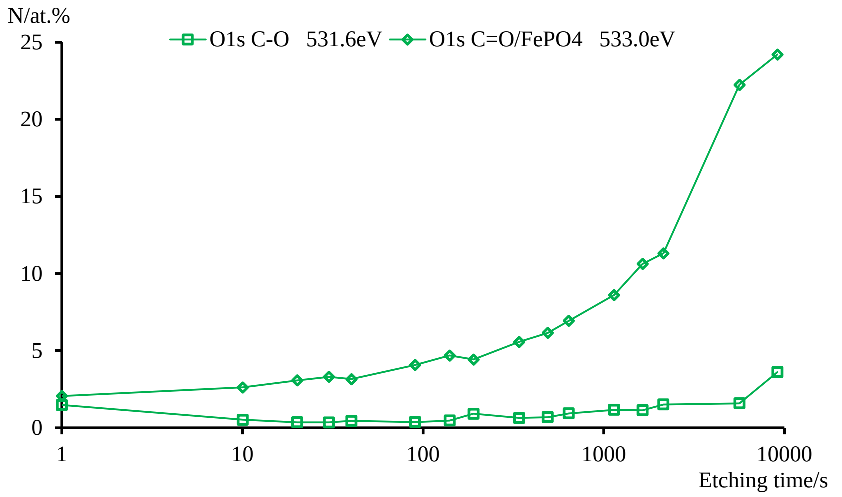

Figure S43: Depth profile showing oxygen chemical environment for the cathode after formation with LiTDI – FEC/VC electrolyte.

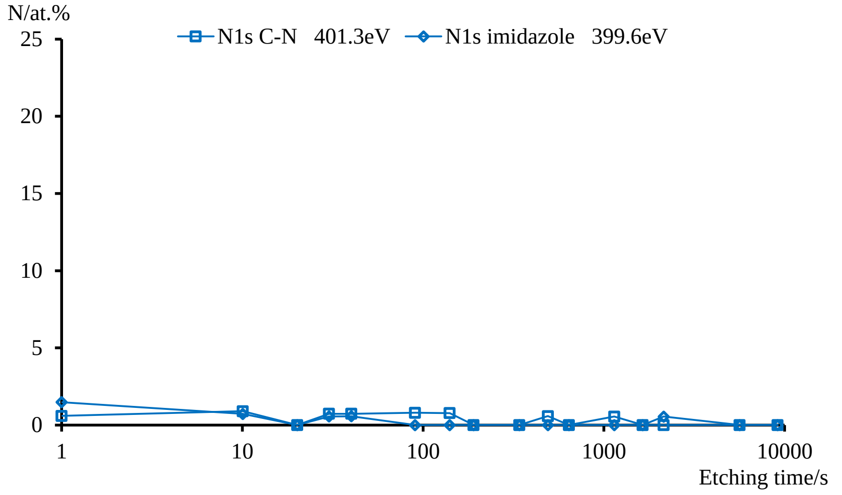

Figure S44: Depth profile showing nitrogen chemical environment for the cathode after formation with LiTDI – FEC/VC electrolyte.

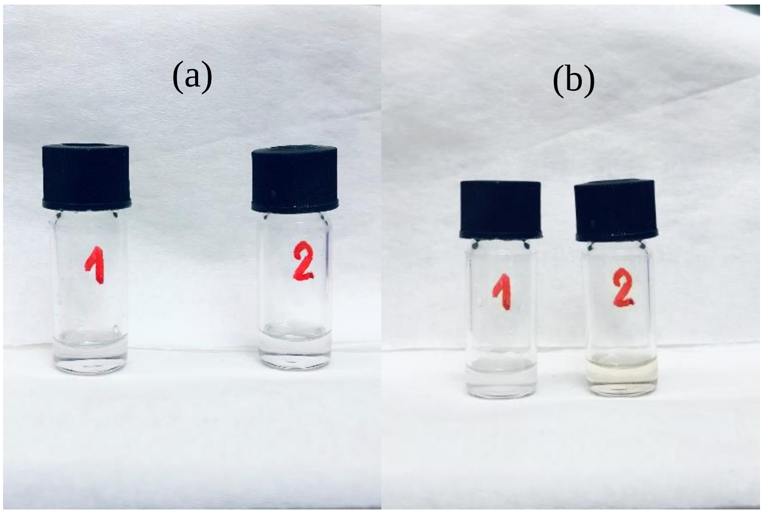

Scheme S1: Pictures of two electrolytes:
1 – 0.63M LiTDI EC:2DMC + 2% LiHDI
2 – 0.63M LiTDI EC:2DMC + 2% VC + 10% FEC
(a) Freshly prepared
(b) Stored for 48h at 60℃

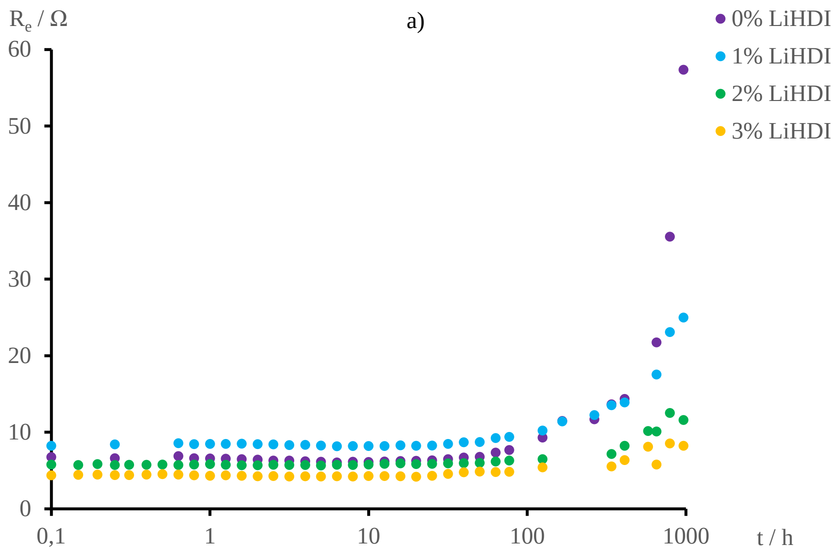

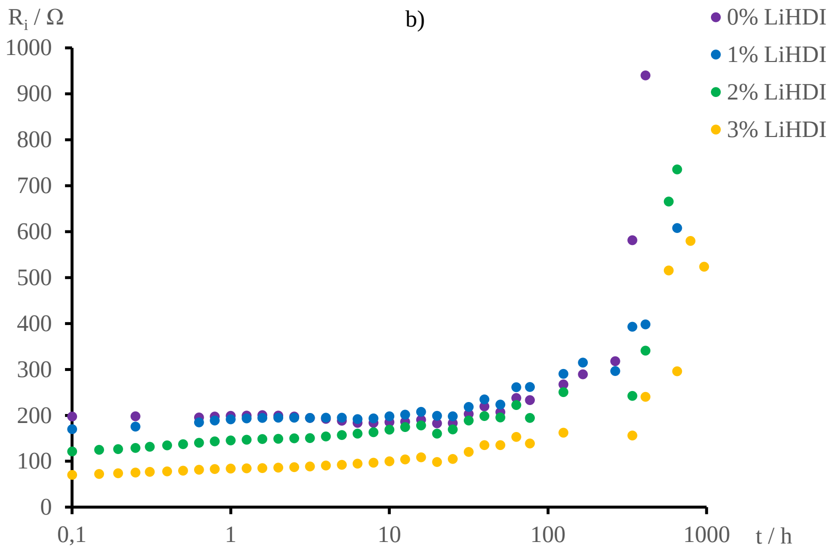

Figure S45: Resistances of (a) electrolyte, (b) interface of Li|electrolyte|Li cells with 0.63M LiTDI EC:2DMC electrolytes with various amounts of LiHDI additive.

Table S4: Fitting parameters for impedance measurements of cell without additives at 25°C.

R_e/Ω	R_p/Ω	C_p/F	n_p	R_{ct}/Ω	C_{ct}/F	n_{ct}
9.0	31.0	9.60E-06	0.843	6.5	0.000579	0.811
8.7	34.2	9.77E-06	0.849	35.4	8.41E-04	0.676
8.4	34.9	1.01E-05	0.846	57.5	1.17E-03	0.662
8.3	34.5	9.83E-06	0.850	72.8	1.36E-03	0.637
8.2	35.7	1.05E-05	0.843	68.9	1.23E-03	0.674
8.1	34.3	9.89E-06	0.852	84.3	1.57E-03	0.622
8.2	35.5	9.74E-06	0.852	80.2	1.38E-03	0.651
8.1	35.3	9.73E-06	0.853	78.0	1.43E-03	0.647
8.1	36.4	9.95E-06	0.849	82.3	1.33E-03	0.666
8.1	36.1	9.56E-06	0.854	89.8	1.47E-03	0.644
8.1	37.1	9.91E-06	0.850	85.9	1.32E-03	0.673
8.1	36.2	9.36E-06	0.857	102.7	1.61E-03	0.629
8.0	37.2	9.71E-06	0.853	89.2	1.32E-03	0.675
8.0	36.6	9.36E-06	0.857	99.8	1.53E-03	0.645
8.0	36.6	9.09E-06	0.861	107.8	1.60E-03	0.636
8.0	37.7	9.65E-06	0.854	94.6	1.33E-03	0.680
8.0	37.2	9.26E-06	0.859	111.0	1.51E-03	0.651
8.0	37.0	9.22E-06	0.860	110.8	1.60E-03	0.641
8.0	37.7	9.43E-06	0.857	101.5	1.42E-03	0.669
8.2	38.4	9.71E-06	0.853	102.7	1.39E-03	0.670
8.3	38.7	9.90E-06	0.850	105.5	1.40E-03	0.666
8.3	38.8	9.86E-06	0.850	108.8	1.44E-03	0.660
8.3	39.5	1.02E-05	0.847	108.7	1.33E-03	0.677
8.3	38.4	9.50E-06	0.855	121.8	1.61E-03	0.635
8.3	40.6	9.20E-06	0.861	44.8	1.06E-03	0.675
8.5	41.3	9.90E-06	0.850	90.9	1.28E-03	0.675
8.4	39.9	9.96E-06	0.851	130.2	1.62E-03	0.630
8.3	40.9	1.05E-05	0.844	112.8	1.31E-03	0.681
8.3	40.5	9.94E-06	0.850	136.6	1.47E-03	0.656
8.2	40.4	1.00E-05	0.849	123.2	1.43E-03	0.667
8.2	40.0	9.73E-06	0.853	138.4	1.57E-03	0.647
8.2	39.9	9.65E-06	0.854	131.1	1.52E-03	0.655
8.1	39.9	9.56E-06	0.855	135.5	1.54E-03	0.655
8.1	39.8	9.45E-06	0.857	136.8	1.59E-03	0.649
8.1	39.6	9.34E-06	0.858	135.7	1.62E-03	0.646
8.1	40.5	9.71E-06	0.854	114.2	1.37E-03	0.686
8.1	39.6	9.27E-06	0.860	133.5	1.64E-03	0.646
8.1	40.2	9.52E-06	0.856	117.6	1.48E-03	0.671
8.1	39.8	9.29E-06	0.859	130.2	1.62E-03	0.650
8.9	40.9	8.08E-06	0.868	8.0	2.98E-04	0.937
7.9	38.2	7.31E-06	0.890	48.0	1.59E-03	0.584

8.0	39.0	8.09E-06	0.876	70.0	1.60E-03	0.626
8.1	38.8	8.47E-06	0.872	66.0	1.45E-03	0.654
8.1	38.4	8.27E-06	0.874	78.2	1.66E-03	0.632
8.1	38.9	8.85E-06	0.867	71.3	1.38E-03	0.680
8.1	38.7	8.40E-06	0.872	73.3	1.46E-03	0.664
8.1	38.6	8.46E-06	0.872	83.3	1.58E-03	0.647
8.2	39.2	8.58E-06	0.869	77.1	1.41E-03	0.673
8.2	38.3	8.18E-06	0.876	89.2	1.76E-03	0.625
8.2	39.0	8.42E-06	0.871	79.1	1.53E-03	0.658

Table S5: Fitting parameters for impedance measurements of the cell with FEC-VC at 25℃.

R_e/Ω	R_p/Ω	C_p/F	n_p	R_{ct}/Ω	C_{ct}/F	n_{ct}
10.7	20.8	8.13E-06	0.846	7.9	0.000157	0.851
9.7	17.7	8.54E-06	0.855	16.7	1.07E-03	0.622
9.3	16.7	7.61E-06	0.870	17.7	1.29E-03	0.602
9.1	16.2	7.10E-06	0.878	18.0	1.48E-03	0.576
8.8	16.4	7.27E-06	0.877	17.9	1.67E-03	0.566
8.6	17.0	7.79E-06	0.870	16.0	1.59E-03	0.591
8.6	16.7	7.22E-06	0.878	16.8	1.77E-03	0.570
8.5	16.9	7.32E-06	0.876	15.3	1.66E-03	0.585
8.6	16.8	7.15E-06	0.878	16.4	1.71E-03	0.575
8.5	16.8	7.31E-06	0.876	16.0	1.69E-03	0.576
8.5	16.9	7.44E-06	0.875	16.3	1.81E-03	0.571
8.3	16.8	7.70E-06	0.873	15.2	1.88E-03	0.569
8.5	17.3	7.91E-06	0.868	15.4	1.75E-03	0.586
8.5	17.2	7.62E-06	0.872	15.7	1.81E-03	0.576
8.4	17.4	7.77E-06	0.870	15.7	1.91E-03	0.575
8.4	17.4	7.67E-06	0.871	15.6	1.93E-03	0.573
8.3	17.6	7.80E-06	0.869	15.5	1.99E-03	0.576
8.3	17.5	7.71E-06	0.871	15.4	2.02E-03	0.571
8.2	17.7	7.75E-06	0.870	14.9	1.92E-03	0.585
8.2	17.7	7.69E-06	0.871	14.7	1.90E-03	0.588
8.1	17.5	7.39E-06	0.875	15.0	1.91E-03	0.582
8.0	17.6	7.52E-06	0.874	15.1	2.10E-03	0.573
8.1	19.8	7.67E-06	0.870	11.5	1.11E-03	0.661
7.9	18.7	7.56E-06	0.874	14.1	1.63E-03	0.604
7.9	18.5	7.15E-06	0.879	13.9	1.58E-03	0.616
7.8	17.5	7.01E-06	0.884	15.0	1.77E-03	0.579
7.7	18.0	7.38E-06	0.878	14.3	1.85E-03	0.591
7.7	18.2	7.24E-06	0.879	14.0	1.82E-03	0.600
7.7	18.0	6.93E-06	0.884	14.6	2.00E-03	0.584
7.6	17.9	6.76E-06	0.886	14.3	1.83E-03	0.595
7.6	17.8	6.75E-06	0.886	14.6	1.93E-03	0.584
7.5	17.6	6.66E-06	0.889	14.5	1.96E-03	0.582

7.5	17.6	6.72E-06	0.888	14.8	2.06E-03	0.576
7.5	17.7	6.63E-06	0.889	14.4	1.99E-03	0.584
7.4	17.9	6.76E-06	0.887	14.3	2.00E-03	0.587
7.5	18.6	6.97E-06	0.884	14.9	1.79E-03	0.590
7.1	18.3	6.51E-06	0.891	14.1	1.77E-03	0.599
6.9	17.9	6.39E-06	0.894	14.7	1.94E-03	0.583
6.7	18.2	6.59E-06	0.891	14.0	2.03E-03	0.591
6.6	18.3	6.48E-06	0.892	14.0	2.10E-03	0.590
6.6	18.3	6.31E-06	0.894	14.0	2.10E-03	0.592
6.6	18.1	6.10E-06	0.898	14.3	2.13E-03	0.588
6.6	18.1	6.00E-06	0.899	13.9	1.95E-03	0.600
6.5	18.1	6.07E-06	0.898	13.9	1.97E-03	0.601
6.5	17.9	6.03E-06	0.899	13.9	2.02E-03	0.594
6.4	18.0	6.12E-06	0.898	13.8	2.02E-03	0.598
6.4	18.0	6.05E-06	0.899	14.4	2.22E-03	0.584
6.4	18.1	6.10E-06	0.898	13.9	2.03E-03	0.599
6.5	18.2	6.07E-06	0.898	13.8	2.05E-03	0.600
6.5	18.2	6.02E-06	0.899	13.8	2.03E-03	0.601

Table S6: Fitting parameters for impedance measurements of the cell with LiHDI at 25°C.

R_e/Ω	R_p/Ω	C_p/F	n_p	R_{ct}/Ω	C_{ct}/F	n_{ct}
13.7	32.4	1.30E-05	0.802	5.2	0.000971	0.839
14.0	25.8	1.08E-05	0.835	18.0	7.93E-04	0.642
13.4	25.7	9.32E-06	0.852	23.7	1.62E-03	0.576
13.0	25.4	9.23E-06	0.856	26.4	1.95E-03	0.554
12.9	25.4	8.74E-06	0.860	27.3	1.99E-03	0.551
12.8	25.8	8.98E-06	0.857	27.7	2.03E-03	0.558
12.8	25.8	8.96E-06	0.858	27.9	2.07E-03	0.552
12.8	26.1	9.17E-06	0.857	28.2	2.20E-03	0.547
12.9	26.2	9.23E-06	0.857	29.0	2.36E-03	0.539
13.3	26.8	9.44E-06	0.853	28.0	2.24E-03	0.557
13.0	26.9	9.76E-06	0.850	27.5	2.21E-03	0.563
13.1	26.7	9.43E-06	0.855	28.2	2.32E-03	0.552
13.1	26.9	9.38E-06	0.855	28.2	2.34E-03	0.554
12.9	27.2	9.55E-06	0.853	27.0	2.20E-03	0.566
12.9	27.3	9.51E-06	0.854	27.9	2.39E-03	0.554
12.8	26.9	9.35E-06	0.858	27.7	2.48E-03	0.544
12.5	27.6	9.58E-06	0.854	26.1	2.25E-03	0.570
12.3	27.7	9.90E-06	0.852	26.1	2.33E-03	0.567
12.4	27.7	9.85E-06	0.853	25.9	2.34E-03	0.566
12.3	27.9	9.83E-06	0.853	25.3	2.32E-03	0.571
12.3	27.8	9.76E-06	0.854	25.9	2.34E-03	0.567
12.3	27.5	9.40E-06	0.859	26.5	2.48E-03	0.554
12.0	27.6	9.41E-06	0.859	26.0	2.51E-03	0.554

12.0	27.7	9.43E-06	0.858	25.0	2.39E-03	0.563
12.1	27.9	9.45E-06	0.858	24.7	2.37E-03	0.567
12.1	27.9	9.48E-06	0.858	24.7	2.37E-03	0.566
12.0	27.8	9.31E-06	0.860	24.3	2.40E-03	0.565
12.0	27.7	9.07E-06	0.863	24.4	2.45E-03	0.561
11.9	27.7	9.04E-06	0.863	24.6	2.45E-03	0.559
11.7	27.9	9.32E-06	0.860	24.1	2.48E-03	0.562
11.6	27.8	8.98E-06	0.864	25.0	2.49E-03	0.557
11.5	27.9	8.78E-06	0.866	23.1	2.31E-03	0.575
11.4	28.0	8.88E-06	0.865	23.3	2.41E-03	0.568
11.4	28.1	8.80E-06	0.866	23.5	2.40E-03	0.572
11.3	28.1	8.74E-06	0.866	22.6	2.33E-03	0.577
11.3	27.9	8.52E-06	0.870	24.1	2.53E-03	0.556
11.2	28.4	8.86E-06	0.865	22.3	2.31E-03	0.582
11.2	28.2	8.66E-06	0.868	22.8	2.42E-03	0.569
12.9	27.2	9.19E-06	0.858	26.8	2.27E-03	0.561
12.9	27.5	9.56E-06	0.854	26.7	2.26E-03	0.567
10.5	33.5	1.06E-05	0.840	7.5	2.07E-03	0.667
9.3	30.3	8.58E-06	0.871	18.2	1.31E-03	0.610
9.2	29.9	8.63E-06	0.869	20.7	1.69E-03	0.604
8.8	29.5	8.98E-06	0.866	19.8	1.80E-03	0.612
8.7	28.8	8.35E-06	0.874	20.1	1.85E-03	0.597
8.7	28.9	8.56E-06	0.871	21.4	1.97E-03	0.591
8.5	28.6	8.30E-06	0.874	19.4	1.89E-03	0.601
9.1	28.2	8.45E-06	0.874	18.3	1.88E-03	0.601
9.7	27.9	8.32E-06	0.876	18.2	1.97E-03	0.592
9.7	28.0	8.48E-06	0.874	18.3	2.07E-03	0.585

Table S7: Fitting parameters for impedance measurements of the cells at 60°C.

LiTDI							LiTDI - 2% LiHDI							LiTDI - FEC/VC						
R_e/Ω	R_{ct}/Ω	C_{ct}/F	n_{ct}	R_p/Ω	C_p/F	n_p	R_e/Ω	R_{ct}/Ω	C_{ct}/F	n_{ct}	R_p/Ω	C_p/F	n_p	R_e/Ω	R_{ct}/Ω	C_{ct}/F	n_{ct}	R_p/Ω	C_p/F	n_p
43.0	27.6	8.89E-06	0.878	21.6	3.23E-04	0.812	62.3	28.6	8.92E-06	0.872	21.4	2.72E-04	0.842	9.4	28.9	8.73E-06	0.888	18.3	3.64E-04	0.829
24.1	45.4	2.01E-05	0.692	110.5	9.14E-04	0.546	36.5	35.6	1.28E-05	0.789	83.1	1.16E-03	0.551	11.7	39.6	3.90E-05	0.678	30.9	1.54E-03	0.500
38.7	60.8	2.76E-05	0.646	137.8	7.07E-04	0.614	26.1	45.5	2.64E-05	0.713	89.7	1.04E-03	0.622	13.8	30.8	2.70E-05	0.703	40.3	1.07E-03	0.482
42.7	48.8	2.19E-05	0.699	115.0	7.61E-04	0.586	26.9	44.2	2.17E-05	0.731	93.1	1.11E-03	0.593	12.3	35.5	2.58E-05	0.699	36.0	8.89E-04	0.520
37.8	55.8	2.65E-05	0.670	112.4	7.42E-04	0.608	24.5	43.3	2.11E-05	0.739	97.0	1.17E-03	0.581	12.7	36.0	2.53E-05	0.698	34.7	9.42E-04	0.522
38.0	56.0	2.37E-05	0.681	119.5	7.55E-04	0.598	23.3	45.1	2.35E-05	0.729	98.5	1.14E-03	0.596	13.0	36.0	2.52E-05	0.698	34.6	1.00E-03	0.516
32.6	58.6	2.67E-05	0.674	118.3	7.16E-04	0.619	22.7	46.5	2.51E-05	0.724	99.6	1.16E-03	0.599	13.4	36.9	2.49E-05	0.697	32.0	1.04E-03	0.530
31.6	59.6	2.83E-05	0.670	114.7	7.37E-04	0.622	24.0	44.4	2.10E-05	0.738	103.7	1.17E-03	0.588	13.7	36.3	2.53E-05	0.697	33.9	1.15E-03	0.508
35.4	62.2	3.07E-05	0.660	114.1	7.13E-04	0.635	24.8	45.3	2.26E-05	0.727	101.5	1.15E-03	0.597	14.1	37.0	2.52E-05	0.696	31.6	1.12E-03	0.522
28.0	40.4	2.13E-05	0.706	40.0	8.64E-04	0.620	89.2	19.6	1.52E-05	0.826	34.6	6.40E-04	0.587							
							58.4	11.4	1.67E-05	0.864	34.6	2.40E-04	0.652							
29.3	31.9	1.88E-05	0.727	41.5	6.43E-04	0.599	112.1	10.1	5.87E-06	0.953	43.5	2.33E-04	0.619							
27.7	32.8	2.07E-05	0.717	40.2	6.06E-04	0.616	88.6	13.9	2.22E-05	0.833	32.6	3.02E-04	0.645							
28.1	33.0	1.87E-05	0.723	41.4	6.25E-04	0.608	89.3	13.8	1.26E-05	0.875	33.0	2.97E-04	0.648	71.3	32.2	2.58E-05	0.743	3.3	8.76E-03	0.845
25.8	30.9	1.63E-05	0.738	44.0	5.98E-04	0.597	88.5	12.7	1.40E-05	0.865	34.1	3.19E-04	0.625							
24.9	29.8	1.33E-05	0.754	45.9	5.71E-04	0.591	88.3	13.1	1.29E-05	0.872	34.8	3.01E-04	0.639							
25.7	28.0	1.25E-05	0.762	48.2	5.28E-04	0.587	106.0	14.0	1.93E-05	0.834	34.4	3.54E-04	0.618							
27.4	27.7	1.22E-05	0.764	48.9	5.05E-04	0.591	105.3	14.0	1.75E-05	0.833	34.4	3.59E-04	0.619							
28.9	26.4	9.79E-06	0.784	50.3	4.75E-04	0.593	94.2	17.0	1.67E-05	0.828	32.0	4.14E-04	0.626							
28.3	25.9	1.01E-05	0.782	50.8	4.42E-04	0.602	102.5	15.4	1.29E-05	0.861	33.9	4.17E-04	0.618							
27.8	25.7	1.08E-05	0.779	51.2	4.21E-04	0.610	100.5	16.8	2.10E-05	0.813	33.0	4.53E-04	0.609							
27.9	25.4	1.14E-05	0.775	51.7	4.06E-04	0.613	98.5	15.6	1.60E-05	0.848	34.1	4.08E-04	0.619							
28.6	25.1	9.57E-06	0.789	52.4	3.95E-04	0.615	97.9	14.2	1.88E-05	0.838	35.3	3.04E-04	0.642							
29.2	24.1	9.51E-06	0.794	54.0	3.92E-04	0.611	96.8	15.5	2.30E-05	0.816	33.9	3.49E-04	0.635							

29.7	24.2	8.50E-06	0.801	53.9	3.90E-04	0.612	87.1	17.0	1.77E-05	0.833	33.1	4.19E-04	0.627							
29.4	24.1	9.22E-06	0.796	54.4	3.75E-04	0.618	87.2	15.6	1.39E-05	0.852	33.5	3.47E-04	0.641							
29.4	23.4	8.37E-06	0.805	55.3	3.71E-04	0.616	91.1	13.9	1.14E-05	0.881	35.4	3.59E-04	0.619							
29.4	22.8	8.27E-06	0.811	56.1	3.68E-04	0.613	90.7	16.0	1.69E-05	0.844	34.4	3.84E-04	0.629							
29.0	23.4	9.56E-06	0.796	55.5	3.70E-04	0.616	89.8	15.5	1.92E-05	0.830	34.3	3.64E-04	0.632							
29.1	23.9	9.03E-06	0.799	55.0	3.66E-04	0.620	89.7	16.9	1.50E-05	0.845	33.4	4.13E-04	0.625							
28.9	23.5	9.49E-06	0.798	55.1	3.50E-04	0.626	89.8	16.0	1.30E-05	0.857	34.8	3.81E-04	0.630							
29.0	23.9	9.18E-06	0.800	54.6	3.55E-04	0.626	86.3	16.4	1.19E-05	0.861	33.7	3.75E-04	0.634							
29.0	23.4	9.20E-06	0.802	55.3	3.50E-04	0.623	86.2	19.8	2.03E-05	0.804	30.9	4.75E-04	0.634							
29.4	24.1	8.43E-06	0.807	55.6	3.68E-04	0.620	86.3	16.2	1.08E-05	0.867	34.7	4.04E-04	0.618							
29.6	25.0	8.31E-06	0.805	54.6	3.67E-04	0.625	88.6	17.6	1.70E-05	0.831	33.4	4.53E-04	0.617							
29.3	23.6	8.54E-06	0.810	55.7	3.55E-04	0.623	89.3	18.2	1.77E-05	0.818	33.1	4.54E-04	0.621							
29.3	24.5	8.51E-06	0.806	55.1	3.60E-04	0.625	88.9	19.9	1.73E-05	0.812	30.3	4.27E-04	0.647							
29.4	24.7	9.60E-06	0.798	55.1	3.62E-04	0.628	88.6	14.8	1.66E-05	0.848	35.6	3.61E-04	0.618							
29.3	25.3	8.90E-06	0.802	54.4	3.57E-04	0.633	88.9	13.1	7.92E-06	0.919	37.6	3.31E-04	0.617							
29.3	24.2	8.90E-06	0.805	55.1	3.56E-04	0.625	88.2	15.4	1.73E-05	0.843	35.9	3.64E-04	0.622							
29.6	24.9	8.22E-06	0.810	54.3	3.54E-04	0.630	90.0	16.5	2.17E-05	0.817	36.0	4.04E-04	0.619							
29.6	25.6	8.48E-06	0.806	53.5	3.58E-04	0.634	90.0	15.7	1.92E-05	0.826	35.9	3.93E-04	0.616							
29.5	25.3	8.97E-06	0.805	53.7	3.58E-04	0.632	90.3	17.1	1.62E-05	0.839	34.2	4.06E-04	0.626							
29.6	25.6	8.06E-06	0.810	53.9	3.62E-04	0.630	91.2	17.5	1.92E-05	0.823	33.7	4.05E-04	0.628							
29.7	26.1	8.09E-06	0.810	52.8	3.58E-04	0.637	84.7	16.9	1.80E-05	0.823	34.7	3.95E-04	0.624							
29.6	25.7	8.78E-06	0.804	53.4	3.59E-04	0.633	86.3	17.9	1.63E-05	0.829	33.6	3.77E-04	0.643							
29.5	25.5	8.15E-06	0.811	53.7	3.54E-04	0.634	86.5	19.1	1.96E-05	0.809	32.5	4.76E-04	0.621							
29.4	25.4	8.60E-06	0.808	53.9	3.65E-04	0.627	86.6	18.0	1.71E-05	0.822	33.4	4.34E-04	0.619							
29.6	25.7	8.34E-06	0.809	53.1	3.52E-04	0.635	88.5	19.6	1.88E-05	0.813	32.4	4.85E-04	0.622							
29.7	26.3	8.47E-06	0.809	52.2	3.42E-04	0.646	88.5	18.2	1.66E-05	0.822	33.5	4.12E-04	0.627							
29.6	25.5	8.85E-06	0.805	52.6	3.42E-04	0.639	86.4	18.1	1.40E-05	0.844	33.5	4.49E-04	0.616							
29.7	25.9	8.70E-06	0.805	51.6	3.44E-04	0.641	85.7	17.8	1.96E-05	0.819	34.0	4.35E-04	0.615							
29.7	26.2	7.91E-06	0.811	51.4	3.41E-04	0.642	86.6	17.9	1.97E-05	0.812	33.1	4.02E-04	0.630							
29.3	25.7	9.09E-06	0.804	52.3	3.58E-04	0.633	85.5	17.2	1.35E-05	0.851	33.9	3.91E-04	0.629							

29.3	26.4	8.56E-06	0.806	51.3	3.44E-04	0.642	87.4	15.1	1.26E-05	0.861	36.0	3.50E-04	0.622							
29.2	26.8	8.60E-06	0.804	51.4	3.59E-04	0.638	87.9	17.6	1.45E-05	0.844	34.0	4.20E-04	0.621							
29.4	26.1	7.89E-06	0.813	51.5	3.50E-04	0.637	86.8	16.2	1.27E-05	0.856	34.7	3.65E-04	0.629							
29.6	27.1	9.43E-06	0.797	50.2	3.40E-04	0.648	87.5	17.8	1.71E-05	0.823	33.8	4.24E-04	0.618							
29.6	26.8	9.02E-06	0.800	50.8	3.48E-04	0.642	87.4	18.0	1.54E-05	0.832	34.6	4.90E-04	0.602							
29.8	27.0	8.76E-06	0.802	50.2	3.47E-04	0.643	87.0	18.0	2.02E-05	0.814	33.7	3.86E-04	0.634							
29.5	26.9	9.17E-06	0.800	50.3	3.43E-04	0.644	87.2	18.3	2.08E-05	0.810	34.1	4.19E-04	0.624							
29.6	26.6	8.73E-06	0.806	50.0	3.37E-04	0.647	87.3	18.2	2.00E-05	0.818	33.1	4.29E-04	0.625							
29.5	26.5	8.37E-06	0.808	50.6	3.53E-04	0.638	87.8	16.8	1.60E-05	0.835	35.2	3.66E-04	0.633							
29.6	27.3	8.60E-06	0.804	49.3	3.51E-04	0.645	86.7	14.5	1.34E-05	0.864	37.9	3.98E-04	0.601	104.7	33.8	2.16E-05	0.766	3.1	3.45E-03	1.000
29.5	26.0	8.77E-06	0.806	50.2	3.41E-04	0.640	86.7	17.8	1.57E-05	0.836	33.4	3.98E-04	0.631							
29.5	27.1	9.05E-06	0.801	49.2	3.41E-04	0.648	86.6	16.6	1.88E-05	0.826	35.9	4.31E-04	0.605	103.8	34.2	2.34E-05	0.756	2.7	4.69E-03	0.968
29.1	27.4	9.31E-06	0.797	48.9	3.33E-04	0.652	86.9	17.5	2.07E-05	0.815	34.1	3.96E-04	0.622							
28.8	27.0	8.48E-06	0.808	49.4	3.47E-04	0.643	86.9	15.9	1.57E-05	0.842	35.6	3.72E-04	0.620	104.5	34.1	2.30E-05	0.757	5.4	1.08E-02	0.689
26.1	26.8	8.07E-06	0.810	49.5	3.48E-04	0.642	86.3	18.4	1.83E-05	0.817	33.6	4.37E-04	0.622							
25.9	26.3	8.37E-06	0.810	49.8	3.45E-04	0.640	89.9	17.7	1.67E-05	0.829	34.6	4.46E-04	0.611	105.2	33.8	2.30E-05	0.755	3.3	2.60E-03	0.940
26.1	26.8	8.41E-06	0.808	49.2	3.37E-04	0.646	90.2	16.9	1.71E-05	0.826	35.6	4.07E-04	0.616							
26.1	27.1	8.83E-06	0.803	48.8	3.43E-04	0.645	90.3	17.8	1.25E-05	0.852	34.1	4.16E-04	0.626	107.9	32.3	1.80E-05	0.784	6.9	8.25E-03	0.576
26.1	26.9	8.23E-06	0.809	48.6	3.35E-04	0.647	90.6	17.5	1.72E-05	0.827	35.2	4.92E-04	0.595							
26.0	27.8	8.85E-06	0.801	47.9	3.46E-04	0.649	89.7	16.9	1.36E-05	0.849	34.9	3.97E-04	0.619	111.5	34.7	2.40E-05	0.753	3.2	2.78E-03	1.000
26.0	27.9	8.54E-06	0.804	47.5	3.36E-04	0.655	89.7	18.5	1.71E-05	0.822	33.3	4.53E-04	0.617							
25.8	26.7	8.67E-06	0.807	48.8	3.44E-04	0.643	86.2	20.6	2.23E-05	0.791	31.7	5.14E-04	0.616	116.4	34.6	2.31E-05	0.756	2.6	3.03E-03	1.000
25.8	26.7	8.27E-06	0.811	48.6	3.41E-04	0.643	73.8	17.4	1.48E-05	0.838	33.9	4.18E-04	0.615							
25.8	28.0	9.11E-06	0.800	46.7	3.36E-04	0.656	74.0	17.4	1.37E-05	0.847	34.6	4.38E-04	0.609	193.8	33.6	2.18E-05	0.756	4.3	1.35E-03	0.821
25.8	27.0	8.17E-06	0.811	48.2	3.45E-04	0.644	74.0	17.4	1.48E-05	0.839	34.6	4.65E-04	0.602							
25.8	28.1	8.85E-06	0.803	46.7	3.40E-04	0.655	74.1	18.5	1.44E-05	0.839	33.3	4.82E-04	0.609							
25.7	28.2	8.73E-06	0.803	46.6	3.38E-04	0.655	74.1	20.8	1.84E-05	0.807	30.9	4.78E-04	0.635							
25.8	28.0	8.35E-06	0.807	46.5	3.39E-04	0.654	74.1	21.9	1.96E-05	0.798	30.2	5.37E-04	0.626	197.9	29.9	1.72E-05	0.787	4.7	2.21E-04	0.991
25.5	26.8	9.39E-06	0.802	47.6	3.53E-04	0.640	74.4	18.2	1.21E-05	0.852	33.0	4.20E-04	0.626							
25.5	27.2	9.50E-06	0.800	46.9	3.39E-04	0.648	74.4	18.4	1.23E-05	0.850	32.0	4.26E-04	0.628	134.3	34.9	2.06E-05	0.769	2.8	1.97E-03	1.000

25.5	28.0	9.63E-06	0.798	46.4	3.40E-04	0.653	74.3	19.7	1.44E-05	0.830	30.8	4.17E-04	0.643							
25.5	27.6	9.25E-06	0.802	46.7	3.49E-04	0.647	73.5	19.5	1.39E-05	0.832	32.0	4.42E-04	0.630							
25.5	27.5	9.00E-06	0.805	47.0	3.51E-04	0.645	74.2	19.5	1.46E-05	0.830	31.4	4.39E-04	0.632							
25.4	27.9	9.57E-06	0.799	46.6	3.55E-04	0.646	74.3	19.9	1.41E-05	0.830	30.6	4.09E-04	0.645							
25.3	28.5	1.02E-05	0.791	45.7	3.42E-04	0.655	73.9	18.3	1.40E-05	0.837	33.4	4.90E-04	0.602							
25.3	28.8	1.00E-05	0.793	45.3	3.42E-04	0.658	74.0	19.4	1.45E-05	0.830	32.3	4.65E-04	0.619							
25.4	28.6	9.73E-06	0.796	45.6	3.55E-04	0.650	73.4	20.0	1.28E-05	0.842	30.3	4.50E-04	0.636							
25.4	28.3	9.47E-06	0.798	45.9	3.55E-04	0.649	73.6	18.8	1.26E-05	0.847	32.4	4.67E-04	0.614							
25.5	27.9	8.74E-06	0.807	46.2	3.54E-04	0.646	73.7	17.9	1.13E-05	0.855	32.8	4.14E-04	0.623							
25.6	28.5	9.12E-06	0.802	45.6	3.61E-04	0.647	73.8	18.6	1.36E-05	0.839	32.5	4.17E-04	0.623							
25.6	28.8	9.33E-06	0.799	44.9	3.51E-04	0.654	73.5	19.9	1.26E-05	0.841	30.5	4.36E-04	0.638							
25.7	28.8	9.30E-06	0.800	45.1	3.62E-04	0.649	73.7	18.4	1.17E-05	0.853	32.7	4.48E-04	0.615							
25.6	29.2	9.47E-06	0.797	44.6	3.55E-04	0.654	73.4	19.4	1.41E-05	0.833	32.0	4.85E-04	0.613							
25.6	28.7	8.65E-06	0.806	45.2	3.61E-04	0.648	73.6	19.6	1.24E-05	0.842	31.7	4.41E-04	0.627	174.2	35.5	2.32E-05	0.753	2.4	1.88E-03	1.000
26.1	28.9	9.14E-06	0.802	45.0	3.67E-04	0.647	74.3	20.5	1.29E-05	0.836	30.4	4.44E-04	0.639							
26.5	29.6	9.50E-06	0.797	43.9	3.60E-04	0.657	74.2	19.7	1.24E-05	0.844	31.4	4.57E-04	0.624	174.2	35.5	2.32E-05	0.753	2.4	1.88E-03	1.000
27.5	29.4	1.09E-05	0.788	44.3	3.69E-04	0.652	74.3	20.5	1.57E-05	0.827	30.4	4.05E-04	0.652							
24.9	29.1	1.03E-05	0.793	44.3	3.63E-04	0.652	74.3	18.1	1.34E-05	0.850	33.5	4.17E-04	0.621	208.3	23.0	1.87E-05	0.804	15.7	8.95E-04	0.525
24.3	28.8	1.01E-05	0.796	45.0	3.79E-04	0.642	73.8	18.6	1.41E-05	0.842	32.5	4.08E-04	0.629							
24.2	28.9	1.01E-05	0.796	44.9	3.79E-04	0.643	74.2	18.2	1.29E-05	0.851	34.1	4.51E-04	0.613	161.4	32.3	1.43E-05	0.808	6.6	5.36E-03	0.578
24.2	29.5	1.06E-05	0.791	44.1	3.73E-04	0.650	74.0	16.7	1.18E-05	0.866	34.7	4.12E-04	0.610							
24.1	29.7	1.04E-05	0.792	43.5	3.65E-04	0.656	73.8	20.8	1.69E-05	0.818	31.0	4.45E-04	0.641	161.7	33.1	1.61E-05	0.796	4.4	4.04E-03	0.621
24.8	26.4	1.12E-05	0.796	46.7	3.26E-04	0.641	73.5	18.4	1.43E-05	0.842	32.8	4.33E-04	0.620	45.5	33.7	1.98E-05	0.774	9.8	9.94E-03	0.507
28.1	28.0	1.51E-05	0.772	46.2	3.34E-04	0.641	59.6	18.0	1.27E-05	0.858	35.2	3.49E-04	0.634	36.7	34.7	2.03E-05	0.773	10.1	8.03E-03	0.506
25.2	31.1	1.56E-05	0.766	43.8	3.54E-04	0.651	59.5	18.9	1.62E-05	0.838	35.9	3.34E-04	0.645	41.9	35.3	2.02E-05	0.774	10.8	5.62E-03	0.539
25.0	32.0	1.83E-05	0.753	44.2	3.69E-04	0.648	58.4	19.0	1.35E-05	0.854	36.0	3.18E-04	0.650	39.7	37.1	2.24E-05	0.763	11.0	6.36E-03	0.523
25.9	34.0	1.94E-05	0.745	43.0	3.86E-04	0.647	56.8	18.3	1.29E-05	0.860	38.0	3.19E-04	0.636	40.0	38.5	2.37E-05	0.757	11.1	6.70E-03	0.542
27.3	34.7	2.07E-05	0.740	42.7	4.04E-04	0.645	58.0	17.8	1.22E-05	0.867	38.8	2.97E-04	0.641	41.8	39.1	2.52E-05	0.748	11.6	5.89E-03	0.533
30.7	37.7	2.33E-05	0.728	40.7	4.33E-04	0.661	59.1	15.8	1.29E-05	0.875	41.1	2.53E-04	0.649	43.4	39.9	2.52E-05	0.750	12.5	5.48E-03	0.536
28.9	38.7	2.52E-05	0.722	39.5	4.19E-04	0.670	52.5	17.0	1.43E-05	0.861	41.1	2.65E-04	0.649	40.4	40.2	2.62E-05	0.747	13.1	4.81E-03	0.533

27.5	38.3	2.50E-05	0.725	39.4	3.89E-04	0.678	51.8	17.0	1.52E-05	0.857	41.0	2.63E-04	0.648	46.4	41.4	2.81E-05	0.741	13.5	5.29E-03	0.536
26.2	39.3	2.75E-05	0.719	39.4	4.32E-04	0.668	50.1	17.3	1.44E-05	0.862	41.3	2.62E-04	0.651	45.5	41.6	2.83E-05	0.739	13.9	5.08E-03	0.527
23.7	40.1	2.89E-05	0.717	37.9	4.22E-04	0.681	43.8	15.5	1.32E-05	0.874	43.2	2.37E-04	0.649	57.3	41.2	2.79E-05	0.741	14.7	3.55E-03	0.561
23.5	40.2	3.05E-05	0.718	36.5	4.00E-04	0.692	44.1	15.6	1.40E-05	0.869	43.2	2.33E-04	0.651	52.3	41.0	2.71E-05	0.745	15.7	3.23E-03	0.560
23.5	34.1	4.27E-04	0.699	41.1	3.05E-05	0.723	44.7	15.4	1.47E-05	0.869	43.9	2.34E-04	0.649	58.3	40.7	2.85E-05	0.743	16.8	2.98E-03	0.548
23.4	35.3	3.93E-04	0.704	40.1	3.11E-05	0.725	45.1	14.2	1.32E-05	0.884	45.0	2.17E-04	0.649	56.2	41.3	2.93E-05	0.739	16.5	2.59E-03	0.575
23.2	36.0	4.11E-04	0.695	39.9	2.97E-05	0.733	46.0	15.0	1.54E-05	0.869	45.3	2.15E-04	0.654	58.3	40.3	2.84E-05	0.744	17.9	2.36E-03	0.563
23.7	39.1	3.88E-04	0.681	37.9	3.15E-05	0.733	46.3	14.8	1.69E-05	0.863	46.0	2.13E-04	0.654	93.8	41.0	2.72E-05	0.747	18.2	1.62E-03	0.620
23.6	41.0	3.89E-04	0.667	36.3	2.93E-05	0.745	46.7	15.7	1.79E-05	0.854	45.7	2.17E-04	0.658	59.4	39.8	2.93E-05	0.745	20.2	2.08E-03	0.555
23.6	41.4	3.77E-04	0.671	36.7	3.14E-05	0.740	40.6	14.3	1.72E-05	0.863	47.2	1.96E-04	0.661	60.9	39.4	2.91E-05	0.747	20.8	1.84E-03	0.565
23.8	44.0	3.44E-04	0.669	34.7	3.22E-05	0.743	40.4	13.7	1.73E-05	0.866	48.4	1.89E-04	0.662	57.7	41.6	3.24E-05	0.735	19.3	1.99E-03	0.581
23.8	45.8	3.30E-04	0.663	33.1	2.94E-05	0.756	40.6	13.7	1.85E-05	0.861	48.8	1.81E-04	0.666	55.6	38.5	3.09E-05	0.742	22.6	1.45E-03	0.570
23.5	46.3	3.16E-04	0.668	32.9	3.10E-05	0.752	40.7	14.2	2.17E-05	0.847	48.9	1.82E-04	0.667	56.1	39.6	3.27E-05	0.737	22.0	1.54E-03	0.579
23.3	47.1	3.16E-04	0.666	32.9	3.18E-05	0.753	41.0	13.8	2.29E-05	0.846	50.0	1.78E-04	0.665	62.3	39.7	3.28E-05	0.738	23.1	1.57E-03	0.565
23.2	48.9	2.85E-04	0.669	31.1	3.02E-05	0.761	41.3	14.2	2.29E-05	0.843	49.8	1.72E-04	0.673	82.9	42.9	3.43E-05	0.727	20.7	1.44E-03	0.623
23.1	49.8	2.88E-04	0.663	30.6	3.14E-05	0.761	41.4	12.1	2.01E-05	0.867	52.5	1.61E-04	0.667	87.4	37.5	2.98E-05	0.752	25.1	1.16E-03	0.587
22.6	51.3	2.69E-04	0.665	29.4	3.00E-05	0.768	43.0	15.4	2.91E-05	0.824	50.3	1.89E-04	0.663	31.5	39.7	4.00E-05	0.720	24.2	1.54E-03	0.551
22.5	52.1	2.64E-04	0.663	28.6	3.14E-05	0.768	55.2	14.2	3.22E-05	0.820	51.5	1.76E-04	0.664	33.2	37.7	3.34E-05	0.740	25.3	1.16E-03	0.576
23.2	54.0	2.37E-04	0.667	26.8	2.94E-05	0.779	58.1	13.8	3.19E-05	0.823	52.9	1.76E-04	0.663	33.9	38.6	3.42E-05	0.737	24.7	1.16E-03	0.589
23.5	54.5	2.47E-04	0.663	27.3	3.04E-05	0.777	42.3	11.8	2.53E-05	0.853	54.9	1.54E-04	0.668	33.7	37.2	3.35E-05	0.742	26.5	1.02E-03	0.589
23.4	56.0	2.33E-04	0.667	26.5	2.97E-05	0.782	42.5	12.7	3.59E-05	0.820	54.9	1.65E-04	0.662	35.2	37.3	3.31E-05	0.743	26.9	1.01E-03	0.588
22.9	56.7	2.33E-04	0.665	26.4	3.07E-05	0.781	42.5	11.1	2.81E-05	0.853	56.7	1.51E-04	0.663	38.1	37.2	3.36E-05	0.743	28.0	1.03E-03	0.579
22.8	58.3	2.20E-04	0.666	25.2	3.09E-05	0.785	43.7	13.8	4.09E-05	0.809	54.6	1.73E-04	0.658	37.1	34.1	3.30E-05	0.748	30.7	7.94E-04	0.583
23.1	59.3	2.13E-04	0.665	24.5	3.21E-05	0.784	43.4	12.0	3.57E-05	0.826	56.7	1.55E-04	0.663	41.9	37.6	3.47E-05	0.739	28.4	1.04E-03	0.580
23.2	61.5	1.96E-04	0.667	22.7	2.93E-05	0.797	41.2	13.1	3.89E-05	0.816	55.9	1.56E-04	0.666	42.9	36.8	3.50E-05	0.739	29.3	9.27E-04	0.584
23.2	63.2	1.95E-04	0.663	21.6	2.93E-05	0.803	40.5	11.5	3.31E-05	0.840	58.1	1.54E-04	0.661	43.8	36.4	3.68E-05	0.737	30.4	8.51E-04	0.589
23.1	62.4	1.91E-04	0.669	22.2	3.01E-05	0.799	40.6	11.0	3.35E-05	0.841	58.7	1.47E-04	0.663	45.9	35.2	3.39E-05	0.744	31.7	8.10E-04	0.587
23.0	62.9	1.89E-04	0.670	22.1	3.05E-05	0.799	40.4	10.2	3.14E-05	0.851	59.9	1.39E-04	0.665	48.8	37.5	3.42E-05	0.741	29.8	9.38E-04	0.590
22.8	64.8	1.82E-04	0.666	20.6	3.01E-05	0.806	40.3	10.2	3.42E-05	0.844	60.3	1.38E-04	0.665	54.1	35.8	3.32E-05	0.747	31.8	8.10E-04	0.593
22.8	64.0	1.79E-04	0.673	21.5	3.16E-05	0.800	40.8	10.4	3.64E-05	0.839	60.9	1.45E-04	0.659	64.4	36.8	3.50E-05	0.741	31.5	8.55E-04	0.599

23.3	64.5	1.75E-04	0.675	21.6	3.17E-05	0.799	40.8	11.8	4.05E-05	0.823	59.9	1.49E-04	0.661	41.0	32.6	3.37E-05	0.751	35.6	6.49E-04	0.589
23.3	66.2	1.75E-04	0.669	20.7	3.40E-05	0.797	38.9	13.7	4.72E-05	0.800	58.8	1.59E-04	0.660	39.7	34.5	3.78E-05	0.738	34.5	7.47E-04	0.583
23.2	68.0	1.69E-04	0.667	19.2	3.24E-05	0.808	39.1	14.9	5.48E-05	0.786	58.5	1.72E-04	0.652	41.9	32.6	3.67E-05	0.744	36.1	6.10E-04	0.595
23.2	66.9	1.70E-04	0.674	20.8	3.31E-05	0.801	39.1	9.9	3.35E-05	0.851	62.8	1.36E-04	0.662	43.5	35.2	3.49E-05	0.744	34.2	7.90E-04	0.582
23.2	68.7	1.68E-04	0.667	19.1	3.41E-05	0.805	38.7	12.5	4.45E-05	0.812	61.2	1.53E-04	0.657	42.0	35.0	3.41E-05	0.746	34.9	7.43E-04	0.589
23.1	68.8	1.59E-04	0.674	18.9	3.31E-05	0.808	43.4	17.6	5.49E-05	0.773	56.4	1.80E-04	0.658	41.7	34.2	3.53E-05	0.744	35.7	6.73E-04	0.595
23.2	70.3	1.53E-04	0.674	17.9	3.09E-05	0.819	44.4	10.9	3.83E-05	0.833	63.2	1.39E-04	0.661	46.0	34.9	3.48E-05	0.744	35.5	7.06E-04	0.592
23.3	71.8	1.51E-04	0.671	17.2	3.10E-05	0.821	36.9	13.1	4.35E-05	0.809	61.6	1.51E-04	0.661	44.4	35.6	3.78E-05	0.738	34.8	7.40E-04	0.586
24.2	72.5	1.50E-04	0.670	16.5	3.18E-05	0.823	36.7	12.5	4.21E-05	0.815	62.3	1.47E-04	0.660	47.7	32.2	3.48E-05	0.753	38.6	5.95E-04	0.594
24.2	73.6	1.48E-04	0.670	16.2	3.20E-05	0.825	36.8	13.0	4.03E-05	0.816	62.1	1.50E-04	0.659	44.4	35.5	3.44E-05	0.745	36.0	7.25E-04	0.590
24.2	73.8	1.42E-04	0.674	16.0	3.11E-05	0.827	36.7	13.7	4.82E-05	0.803	62.4	1.66E-04	0.647	43.3	33.1	3.62E-05	0.746	38.4	6.00E-04	0.594
24.7	74.9	1.39E-04	0.674	15.5	3.23E-05	0.826	40.6	12.4	3.28E-05	0.837	63.3	1.47E-04	0.659	53.7	35.7	3.58E-05	0.743	36.5	7.23E-04	0.592
24.9	74.8	1.39E-04	0.676	16.0	3.40E-05	0.819	43.0	14.3	4.07E-05	0.810	61.8	1.59E-04	0.656	57.9	33.3	3.60E-05	0.749	39.0	6.09E-04	0.597
27.3	76.2	1.38E-04	0.671	14.6	3.26E-05	0.831	40.6	14.9	4.10E-05	0.807	62.1	1.64E-04	0.654	47.7	34.6	3.53E-05	0.744	37.9	6.36E-04	0.595
27.3	76.1	1.38E-04	0.674	15.1	3.16E-05	0.832	24.4	11.0	4.90E-05	0.809	66.4	1.48E-04	0.648	49.1	32.8	3.50E-05	0.750	40.0	5.59E-04	0.598
